# Characterizing the evolving SARS-CoV-2 seroprevalence in urban and rural Malawi between February 2021 and April 2022: A population-based cohort study

**DOI:** 10.1016/j.ijid.2023.10.020

**Published:** 2023-12

**Authors:** Louis Banda, Antonia Ho, Stephen Kasenda, Jonathan M. Read, Chris Jewell, Alison Price, Estelle McLean, Albert Dube, David Chaima, Lyson Samikwa, Tonney S. Nyirenda, Ellen C. Hughes, Brian J. Willett, Annie Chauma Mwale, Abena S. Amoah, Amelia Crampin

**Affiliations:** 1 Malawi Epidemiology and Intervention Research Unit, Malawi; 2 Medical Research Council-University of Glasgow Centre for Virus Research, Glasgow, United Kingdom; 3 Lancaster University, Lancaster, United Kingdom; 4 London School of Hygiene and Tropical Medicine, London, United Kingdom; 5 Kamuzu University of Health Sciences, Blantyre, Malawi; 6 Public Health Institute of Malawi, Lilongwe, Malawi; 7 Leiden University Medical Center, Leiden, The Netherlands; 8 School of Health and Wellbeing, University of Glasgow, Glasgow, United Kingdom

**Keywords:** SARS-CoV-2, Seroprevalence, Longitudinal cohort, Community, Malawi

## Abstract

**Objectives:**

This study aimed to investigate the changing SARS-CoV-2 seroprevalence and associated health and sociodemographic factors in Malawi between February 2021 and April 2022.

**Methods:**

In total, four 3-monthly serosurveys were conducted within a longitudinal population-based cohort in rural Karonga District and urban Lilongwe, testing for SARS-CoV-2 S1 immunoglobulin (Ig)G antibodies using an enzyme-linked immunosorbent assay. Population seroprevalence was estimated in all and unvaccinated participants. Bayesian mixed-effects logistic models estimated the odds of seropositivity in the first survey, and of seroconversion between surveys, adjusting for age, sex, occupation, location, and assay sensitivity/specificity.

**Results:**

Of the 2005 participants (Karonga, n = 1005; Lilongwe, n = 1000), 55.8% were female and median age was 22.7 years. Between Surveys (SVY) 1 and 4, population-weighted SARS-CoV-2 seroprevalence increased from 26.3% to 89.2% and 46.4% to 93.9% in Karonga and Lilongwe, respectively. At SVY4, seroprevalence did not differ by COVID-19 vaccination status in adults, except for those aged 30+ years in Karonga (unvaccinated: 87.4%, 95% credible interval 79.3-93.0%; two doses: 98.1%, 94.8-99.5%). Location and age were associated with seroconversion risk. Individuals with hybrid immunity had higher SARS-CoV-2 seropositivity and antibody titers, than those infected.

**Conclusion:**

High SARS-CoV-2 seroprevalence combined with low morbidity and mortality indicate that universal vaccination is unnecessary at this stage of the pandemic, supporting change in national policy to target at-risk groups.

## Highlights

• This was a longitudinal SARS-CoV-2 serosurvey in an urban & rural cohort in Malawi.
• Post-Omicron SARS-CoV-2 seroprevalence was very high (rural: 89%; urban: 94%).
• Most SARS-CoV-2 infections were subclinical; few required healthcare attendance.
• Seroconversion risk varied by location & age across the successive infection waves.
• Hybrid immunity was associated with higher seroprevalence and antibody titers.

## Introduction

**Figure 1:**
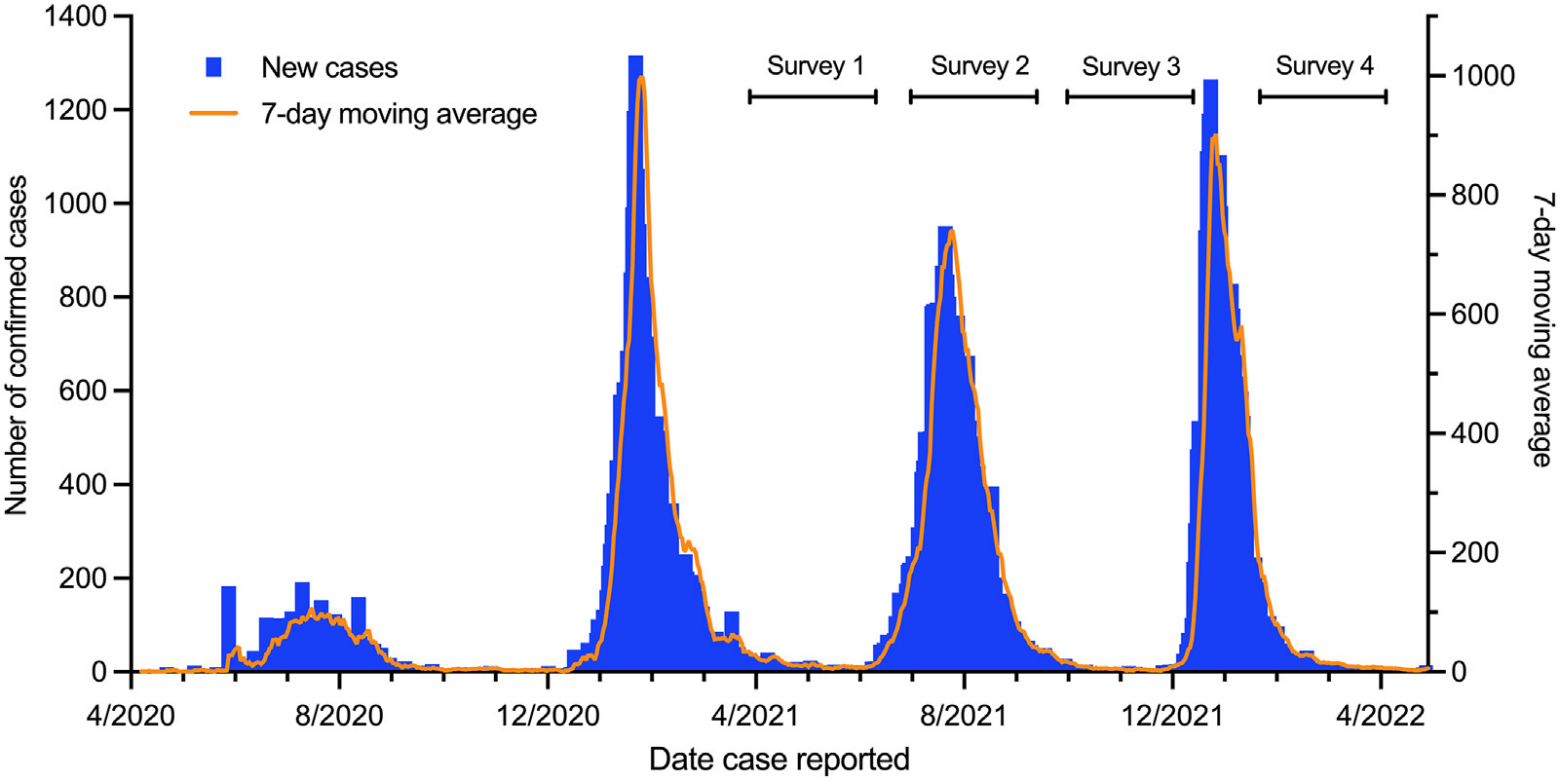
Timing of the serosurvey rounds with national daily new cases of laboratory-confirmed SARS-CoV-2 infection in Malawi Data obtained from the Public Health Institute of Malawi. The four waves were driven by presumed ancestral strain (no sequencing data), Beta, Delta, and Omicron BA1/2 variants respectively. Note the amplitude of the observed peaks of the first and subsequent waves are not comparable due to differing testing capacity during the two time periods.

Malawi, one of the lowest-income countries in Africa [1], has experienced four waves of COVID-19 in the first 2 years of the pandemic; June-August 2020 (wave 1, likely ancestral), December 2020-April 2021 (wave 2, Beta) June-September 2021 (wave 3, Delta) and December 2021-January 2022 (wave 4, Omicron BA.1/2; Figure 1) [2]. Due to limited SARS-CoV-2 testing [3] and surveillance, in addition to a high proportion of asymptomatic infections, the number of confirmed cases likely substantially underestimates the true burden of COVID-19 in Malawi, even more so than in other settings.

The first COVID-19 vaccine doses were delivered on 11 March 2021, initially to key occupational groups and vulnerable adults, rapidly extending to all adults and subsequently adolescents. Only ChAdOx1 nCoV-19 (Oxford AstraZeneca [AZ]) and Ad26.COV2.S (Johnson & Johnson [J&J]) was available before May 2022. By the end of April 2022, 8.4% (n = 1,120,521) of the target population had been vaccinated with two doses of AZ or a single dose of J&J vaccine. A further 1,205,496 (9.1%) were partially vaccinated with a single AZ dose and 4388 had received a booster dose [4].

In Malawi, SARS-CoV-2 seroprevalence studies have relied on convenience samples (e.g. healthcare workers [5,6] and blood donors [7]) and community-based serosurveys have been cross-sectional [8,9]. These approaches provide a limited understanding of the dynamic changes in SARS-CoV-2 antibody responses over time. Longitudinal population-based serological studies are needed to elucidate the spatial and temporal changes in the proportion of the community exposed to SARS-CoV-2 and to characterize how exposure to sequential variants and vaccination impact the magnitude and duration of antibody responses, to inform public health policy.

To address this gap, we investigated SARS-CoV-2 seroprevalence and factors associated with seropositivity using population-based longitudinal data collected in rural and urban communities in Malawi between February 2021 and April 2022.

## Methods

### Study population

We conducted a prospective cohort study on randomly selected households from population-based cohorts operated by the Malawi Epidemiology and Intervention Research Unit (MEIRU): rural (Karonga Health and Demographic Surveillance Site [HDSS], Northern Region; population 51,000) and urban (Area 25, Lilongwe, Central Region; population 75,000) (Supplementary Figure 2). Details on the study sites, sample size, and sampling approach for household selection are included in the Supplementary Material. Participants were recruited between 24 February-8 June 2021 (Survey [SVY] 1; post-Beta wave), with three subsequent 3-monthly follow-ups: SVY2, 28 June-13 September 2021 (during Delta wave); SVY3, 4 October–10 December 2021 (post-Delta wave); and SVY4, 27 January-22 April 2022 (post-Omicron BA.1/2 wave) (Figure 1) [10].

### Study procedures

After establishing written, informed consent for participants or guardians of minors (<15 years) and vulnerable groups (who were assenting), baseline demographics, medical and COVID-19 vaccination history, socioeconomic indicators, COVID-19 related symptoms (since April 2020, and within the previous 2 weeks), prevention behaviors, and recent exposures were collected by interviewer-led questionnaire (see Supplementary Material), with the option of adults answering questions for recruited minors. An additional household socioeconomic questionnaire was administered to the head of each household or other informed member. HIV status was self-reported. At subsequent surveys, reported symptoms (since the last survey, and within the previous 2 weeks), and healthcare-seeking behavior, in addition to updated exposure and vaccination histories were captured. For analysis, two doses of AZ vaccine or one dose of J&J vaccine were categorized as “two doses”.

Venous blood was obtained at each survey and stored at -80°C at the MEIRU laboratories before testing. At the same visit, 50% of randomly selected adult participants were approached for self-collection of combined nasal and throat swabs for SARS-CoV-2 polymerase chain reaction (PCR) testing in SVY1, expanded to all adult participants for SVY2-4.

### SARS-CoV-2 PCR

Swab samples were stored at -80°C and were tested at the Public Health Institute of Malawi laboratories for the presence of SARS-CoV-2 RNA on the Abbott M2000 RT system automated analyzer using the Abbott SARS-CoV-2 assay (Abbott Park, IL, USA), a dual target assay detecting RNA-dependent RNA polymerase (RdRp) and nucleocapsid (N) genes**.** The assay was calibrated with Abbott RealTime SARS-CoV-2 positive and negative quality controls before analyses. Cycle threshold (Ct) values of ≤32 for both targets were interpreted as positive for SARS-CoV-2 RNA.

### SARS-CoV-2 serology

We measured serum SARS-CoV-2 immunoglobulin (Ig)G antibody titers targeting spike (S1) antigen (www.nibsc.org) using an enzyme-linked immunosorbent assay (ELISA) developed at Icahn School of Medicine at Mount Sinai [11]. Raw optical density (OD) values were normalized using the equation, “(mean sample OD–negative control mean)/negative control mean”. Using the *mixtools* package in R [12], we ran a finite mixture model to classify the samples as seropositive or seronegative based on normalized A_450_ values [13,14]. Seropositivity cut-off was defined as the mean of the Gaussian distribution of the seronegative population plus five SDs of the seronegative population. The normalized OD ratio threshold was 1.620. This assay has undergone rigorous validation at the medical research council  (MRC)-University of Glasgow Centre for Virus Research [15]. Additional assay validation using negative and positive control samples from Malawian individuals (see Supplementary Material) estimated an assay sensitivity of 86.5% (95% CI 79.6-93.3%) and specificity of 98.8% (95% CI 97.4-100%) (Supplementary Figure 3).

Seroconversion is defined as conversion from SARS-CoV-2 S1 IgG negative to IgG positive in the subsequent survey, while seroreversion denotes a decline in SARS-CoV-2 S1 IgG antibody titers, such that they become seronegative, from previously being seropositive.

### Statistical analysis

We modeled SARS-CoV-2 seropositivity and seroconversion, with categorical fixed effects (age group, etc*.*), and a random intercept for household membership using a common Bayesian mixed-effects logistic modeling framework. We modeled test sensitivity and specificity using the values provided (we did not estimate these as fitting was unreliable). Three models were fitted to estimate: (1) site- and survey-specific seroprevalence, adjusting for age group and vaccination dose (SVY2-SVY4 only); (2) site- and age-specific cumulative seroconversion risk among unvaccinated and previously seronegative individuals; (3) seroconversion risk between surveys, adjusting for age group, sex, household size, occupation type and site (See Supplementary Material).

For participants who provided sera in all four surveys, we plotted trajectories of normalized OD ratios over time. Additionally, we compared median and interquartile range (IQR) of normalized OD ratios, stratified by serostatus at enrollment (SVY1) and vaccine status using Wilcoxon rank sum test. Binomial CIs were calculated for proportions.

Data analyses were performed using Stata 16.0 (StataCorp, Texas, USA) and R statistical (version 4.0.1).

### Ethical approval

The study was approved by the Malawi College of Medicine Research Ethics Committee (P11/20/3177) and the University of Glasgow College of Medicine, Veterinary and Life Sciences Research Ethics Committee (200200056).

## Results

### Cohort characteristics

We enrolled 285/376 (75.8%) approached households in Karonga HDSS and 343/1024 (33.5%) in Area 25, Lilongwe (Supplementary Figure 1). In total, 2005 participants (Karonga [rural] n = 1005; Lilongwe [urban] n = 1000) consented to participate in the cohort (Table 1).

**Table 1:** Baseline characteristics of the cohort.

	Karonga (rural)			Lilongwe (urban)		
	All (n = 1005)	<15 years (n = 366)	≥15 years ( = 639)	All (n = 1000)	<15 years (n = 291)	≥15 years (n = 709)
**Age group (years)** – median (IQR)	20·2 (11·2-39·2)			25·2 (12·9-40·2)		
<5	64 (6·4)			75 (7·5)		
5-14	302 (30·1)			216 (21·6)		
15-39	401 (40·9)			457 (45·7)		
40-59	178 (17·7)			177 (17·7)		
> = 60	60 (6·0)			75 (7·5)		
**Sex** (Male) – N (%)	506 (50·4)	183 (50·0)	323 (50·6)	381 (38·1)	139 (47·8)	242 (34·1)
**Comorbidities**	n = 1005	n = 366	n = 639	n = 1000	n = 291	n = 709
HIV infection	37 (3·7)	4 (1·1)	33 (5·2)	62 (6·2)	2 (0·7)	60 (8·5)
Asthma	80 (8·0)	30 (8·2)	50 (7·8)	39 (3·9)	8 (2·8)	31 (4·4)
Chronic lung disease (not asthma)	3 (0·3)	2 (0·6)	1 (0·2)	2 (0·2)	0 (0)	2 (0·3)
Heart disease	17 (1·7)	0 (0)	17 (2·7)	4 (0·4)	0 (0)	4 (0·6)
Hypertension	51 (5·1)	0 (0)	51 (8·0)	89 (8·9)	0 (0)	89 (12·6)
Chronic kidney disease	5 (0·5)	0 (0)	5 (0·8)	0 (0·0)	0 (0)	0 (0)
Diabetes	5 (0·5)	0 (0)	5 (0·8)	19 (1·9)	1 (0·3)	13 (5·2)
Tuberculosis (past or current)	7 (0·7)	1 (0·3)	6 (0·9)	16 (1·6)	1 (0·3)	15 (2·1)
Cancer	1 (0·1)	0 (0)	1 (0·2)	3 (0·3)	0 (0)	3 (0·4)
Stroke	5 (0·5)	1 (0·3)	3 (0·6)	2 (0·2)	0 (0)	2 (0·3)
Liver disease	3 (0·3)	1 (0·3)	2 (0·3)	0 (0·0)	0 (0)	0 (0)
**Comorbidities**	n = 1005	n = 366	n = 639	n = 1000	n = 291	n = 709
None	828 (82·4)	330 (90·2)	528 (82·6)	808 (80·6)	279 (95·9)	589 ((83·1)
1	144 (14·3)	33 (9·0)	98 (15·3)	161 (16·1)	12 (4·1)	108 (15·2)
≥2	33 (3·3)	3 (0·8)	13 (2·0)	33 (3·3)	0 (0)	12 (1·7)
**Occupation type**	n = 1005	n = 366	n = 639	n = 1000	n = 291	n = 709
Unwaged	877 (87·3)	366 (100)	511 (80·0)	612 (61·2)	291 (100)	322 (45·2)
Irregular wage/piecework	90 (9·0)	0 (0)	90 (14·1)	235 (23·5)	0 (0)	234 (33·0)
Regular wage	38 (3·8)	0 (0)	38 (6·0)	153 (15·3)	0 (0)	153 (21·6)
**Monthly household income**^a^**(quintile)**	n = 1003			n = 996		
1 (lowest)	238 (23·7)			219 (22·0)		
2	196 (19·5)			221 (22·1)		
3	170 (17·0)			229 (23·0)		
4	199 (19·8)			173 (17·3)		
5 (highest)	200 (19·9)			154 (15·4)		
**Asset index**^b^**(quintile)**	N = 1003			N = 1000		
1 (lowest)	367 (36·5)			223 (22·3)		
2	70 (6·7)			177 (17·7)		
3	185 (18·4)			215 (21·5)		
4	187 (18·6)			186 (19·6)		
5 (highest)	196 (19·5)			199 (19·9)		
**Highest level of education**	n = 1005	n = 366	n = 639	n = 1000	n = 291	n = 709
Never attended school	89 (8·9)	82 (22·4)	7 (1·1)	99 (9·9)	70 (24·1)	29 (4·1)
Primary (1-5 years)	299 (29·8)	212 (57·9)	87 (13·6)	256 (25·6)	169 (58·1)	86 (12·1)
Primary (6-8 years)	363 (36·1)	70 (19·1)	293 (45·9)	191 (19·1)	43 (14·8)	147 (20·7)
Secondary	244 (24·3)	2 (0·6)	243 (38·0)	380 (37·9)	9 (3·1)	371 (52·3)
Tertiary	10 (1·0)	0 (0)	9 (1·4)	76 (7·6)	0 (0)	76 (10·7)
**Households** - N	285			343		
**Household size** – median (IQR)	5 (4-7)			6 (4-7)		
1-3	137 (13·6)			126 (12·6)		
4-5	425 (42·3)			341 (34·0)		
6-7	317 (31·5)			336 (33·5)		
≥8	126 (12·5)			199 (19·9)		
**Crowding index**^c^	N = 1003			n = 996		
<1·5	366 (36·5)			155 (15·7)		
1·5-2·4	459 (45·8)			475 (47·7)		
≥2·5	178 (17·8)			366 (36·8)		
**Workplace (adults only)**	n = 639		n = 639	n = 709		n = 709
Predominantly indoors	150 (23·5)		150 (23·5)	445 (62·8)		445 (62·8)
Predominantly outdoors	489 (76·5)		489 (76·5)	264 (37·2)		264 (37·2)

a Reported total household income in Malawian kwacha was categorized into quintiles by study location.

b A total asset score was calculated for each participant, based on the total mean estimated value of a number of household items. Quintile of asset wealth by study location was generated. See Supplementary Material for further detail.

c Number of household members divided by the number of sleeping rooms in dwelling. IQR, interquartile range.

Median age was higher in Lilongwe (25.2 years, IQR 12.9-40.2) compared to Karonga participants (20.2 years, IQR 11.2-39.2). Fewer participants in Karonga were female (49.6% vs 61.9%). Adult participants (aged ≥15 years) from Karonga were less likely to have waged employment (6.0% vs 21.6%) or to have attended secondary education (39.4% vs 63.0%), compared to Lilongwe. Most lived in multi-occupant households (95.7% Karonga, 91.3% Lilongwe), A higher proportion of Karonga adult participants reported working predominantly outdoors than Lilongwe participants (76.5% vs 37.2%). Of the adults surveyed, 17.4% of Karonga and 16.9% of Lilongwe participants reported one or more long-term health conditions. Self-reported HIV prevalence among adults was 5.2% in Karonga and 8.5% in Lilongwe, which were marginally lower than national figures (Northern Region [including Karonga]: 6.6%, 95% CI 5.3-7.9%; Lilongwe: 10.6%, 95% CI 8.7-12.6%) [16]. Study retention was higher in Karonga; 94.7% (n = 952), 93.6% (n = 941) and 88.5% (n = 889) of the rural cohort took part in SVY2, SVY3 and SVY4, compared to 72.1% (n = 721), 60.3% (n = 603), and 53.1% (n = 531) of the urban cohort. Those who were lost to follow-up in SVY2-SVY4 were more likely to be female (60.5% vs 53.8%) than those who attended all surveys. Attrition was highest among 15-39 years, with only 64.6% (554/858) completing the final survey, compared to 75.6% (497/657) participants aged <15 years and 75.3% (369/490) aged ≥40 years.

### COVID-19 vaccine uptake

The proportion of the study cohort reporting receipt of ≥1 dose of COVID-19 vaccine increased between SVY1-SVY4; from 2.6% (95% CI 1.7-3.8; 26/1005 [SVY1]) to 17.9% (15.4-20.6; 159/889 [SVY4]) in Karonga and 7.6% (6.0-9.4; 76/1000 [SVY1]) to 26.4% (22.7-30.3; 140/531 [SVY4]) in Lilongwe (Supplementary Table 1). Reported vaccine refusals were substantially higher in Karonga (SVY1 to SVY4: 1.4- 17.4%) compared to Lilongwe (0.1-1.1%). Multinomial modeling of vaccination and the number of doses received showed variation by age group and site, with those <30 years being significantly less likely to receive a first or second dose than older individuals, and vaccinated individuals more likely to receive a second dose in Lilongwe (Supplementary Tables 2 & 3).

### SARS-CoV-2 PCR positivity and reported symptoms

SARS-CoV-2 PCR positivity among adult participants was significantly higher in Karonga than Lilongwe in SVY1 (9.7%, 95% CI 6.6-13.6 [29/299] vs 0.7%, 95% CI 0.1-2.6 [2/279]) and SVY2 (9.7%, 7.2-12.6 [48/497] vs 4.4%, 2.6-6.7 [19/436]) (Supplementary Table 4). However, SARS-CoV-2 PCR positivity was low at both sites (<2%) in SVY3 and SVY4, with no difference by location. The proportion of PCR-positive individuals that reported ≥1 COVID-19-associated symptoms within 14 days ranged from 23.1% (5.0-53.8%; 3/13; SVY3) to 68.7% (56.2-79.4; 46/67; SVY2). Participants that were SARS-CoV-2 PCR-positive were more likely to report fever, fatigue, shortness of breath, and diarrhea than SARS-CoV-2 PCR-negative individuals in SVY1 (Supplementary Table 5), and similar symptoms, in addition to chills/shivers, myalgia, cough, runny nose, loss of smell, taste, and appetite predicted SARS-CoV-2 PCR positivity in SVY2.

### SARS-CoV-2 seroprevalence estimates

**Figure 2:**
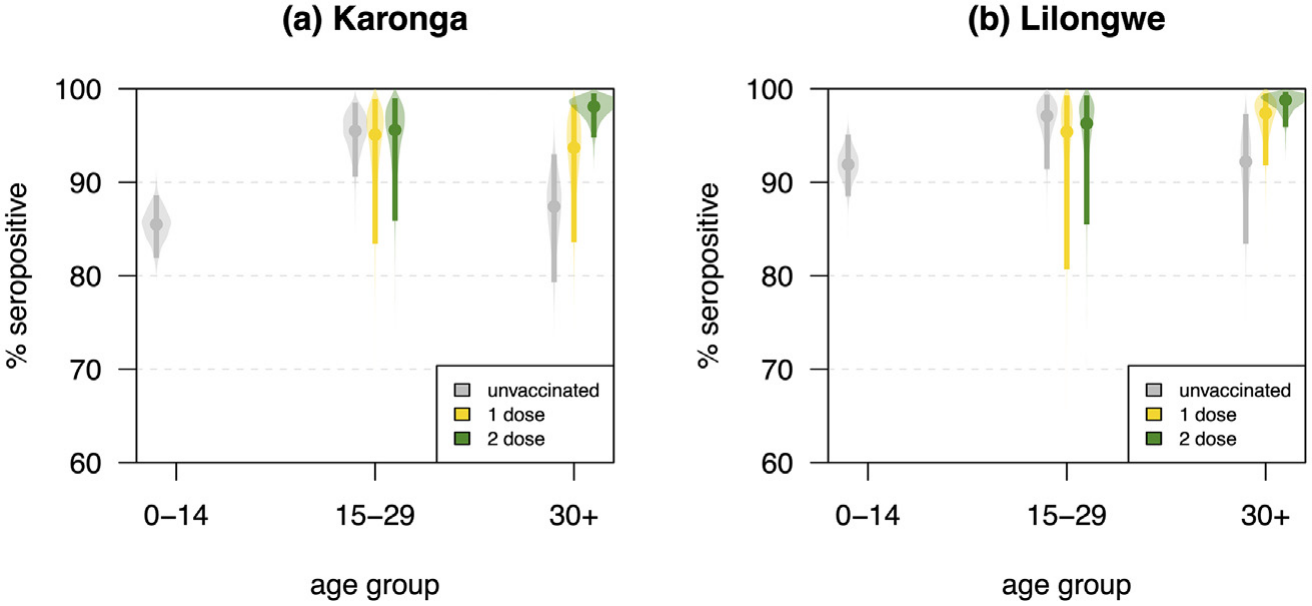
SARS-CoV-2 seroprevalence by age group and vaccine status in (a) Karonga and (b) Lilongwe at Survey 4.

Population-weighted seropositivity at SVY1 was 26.3% (95% credible interval (CrI) 20.7-32.1%) in Karonga and 46.4% (40.2-53.0%) in Lilongwe, increasing to 89.2% (85.4-92.2%) in Karonga and 93.9% (90.6-96.5%) in Lilongwe at SVY4. SARS-CoV-2 seropositivity did not differ by COVID-19 vaccination status among adult participants in Lilongwe, and those aged 15-29 years in Karonga (Figure 2). However, those aged 30+ years vaccinated with two doses in Karonga had significantly higher seroprevalence than unvaccinated individuals (87.4%, 95% CrI 79.3-93.0% [unvaccinated]; 93.7%, 83.6-98.3% [1 dose]; 98.1%, 94.8-99.5% [2 doses]).

**Figure 3:**
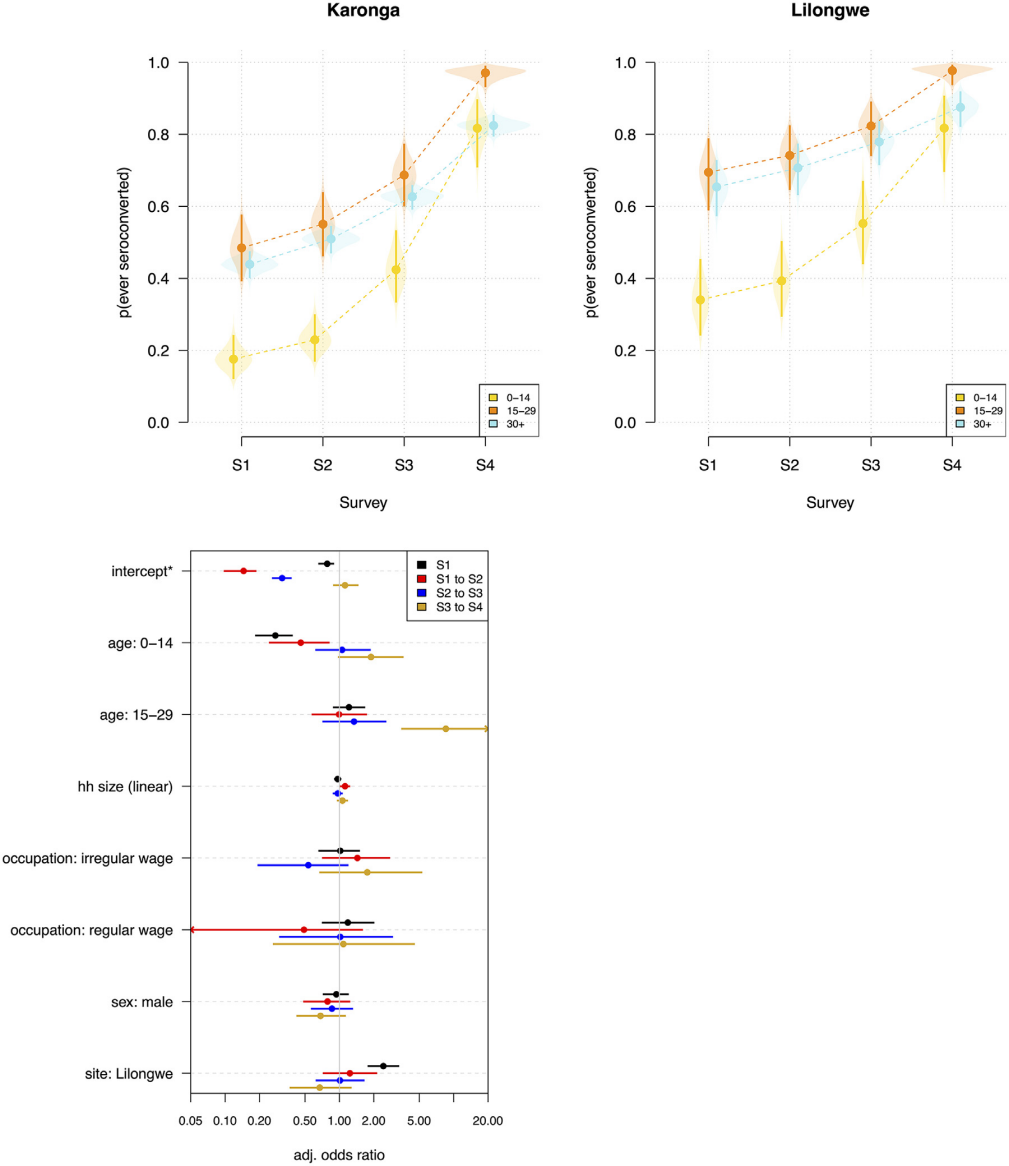
(a) Cumulative probability of ever seroconverting to SARS-CoV-2 over successive surveys, stratified by age group and study site; (b) Association of demographic and other factors with seroconversion among unvaccinated individuals. Note, the values of the intercepts are odds but are plotted on the same scale as the other variables.

Among unvaccinated individuals, SARS-CoV-2 seroprevalence increased over successive surveys at both sites but was consistently highest among those aged 15-29 years (Karonga: 48.5%, 95% CrI 39.2-57.7% [SVY1] to 97.1%, 93.1-99.1% [SVY4]; Lilongwe: 69.5%, 58.9-78.9% [SVY1] to 97.7%, 93.7-99.3% [SVY4]), compared to children <15 years (Karonga: 17.6%, 12.0-24.3% [SVY1] to 81.7%, 70.8-89.8% [SVY4]; Lilongwe: 34.0%, 24.1-45.4% [SVY1] to 81.7%, 69.5-90.8% [SVY4]) (Figure 3a).

A high proportion of SARS-CoV-2 seropositive participants reported no COVID-19 symptoms, ranging from 56.4% (95% CI 53.4-59.4% [SVY4]) to 65.8% (62.4-69.0% [SVY1]). Among seropositive participants who reported ≥1 COVID-19 symptom, 30.5% (25.2-36.2%) attended a health facility as an outpatient and 1.1% (0.2-3.1%) required hospital admission. Healthcare attendance did not differ by SARS-CoV-2 seropositivity in SVY1-3. In SVY4, SARS-CoV-2 seronegative individuals were more likely to report healthcare attendance than seropositive individuals (58.1 vs 42.4%). Over the study period, there were five deaths among Karonga participants (none were consistent with COVID-19) and four in Lilongwe participants (3/4 had preceding symptoms consistent with COVID-19).

### Individual, household-level, and geospatial risk factors for seroconversion

Among unvaccinated participants, the risk of seroconversion decreased and then increased between successive surveys in Karonga (odds in SVY1, 0.78 (95% CrI 0.65-0.90); SVY2 0.15 (0.10-0.19); SVY3, 0.32 (0.26-0.38); SVY4, 1.12 (0.88-1.47); Figure 3b). In SVY1, seropositivity was higher in Lilongwe than Karonga participants (adjusted odds ratio [aOR] 2.42, 95% CrI 1.77-3.33), and lower in children compared to 30+ years adults (aOR 0.28, 0.18-0.39). Children were also at reduced risk of seroconversion between SVY1 and SVY2, compared to 30+ years adults (aOR 0.46, 0.24-0.82). Participants aged 15-29 years were at increased risk of seroconversion between SVY3 and SVY4 compared to 30+ years adults (aOR 8.54, 3.47-24.98). Household size was significantly associated with seroconversion between SVY1 and SVY2 (aOR 1.12, 1.01-1.24), but not between other surveys. Participant sex or occupational status were not associated with seropositivity in SVY1 or seroconversion between surveys (Figure 3b). After adjustment, seroconversion risk was similar between study sites after the Beta wave.

There was no evidence for spatial correlation between households in initial seropositivity (SVY1) or seroconversion between surveys, at scales >50m and ≤3km (Supplementary Figure 4).

### Persistence and trajectory of SARS-CoV-2 antibodies

We evaluated the persistence and trajectory of SARS-CoV-2 antibody responses among the 1264 participants who had sera collected at all four surveys (Karonga n = 841, 83.7%; Lilongwe n = 423, 42.3%). Participants who were seronegative at baseline (S1-) and never vaccinated had the lowest seroprevalence at SVY4 (60.6%; 95% CI 56.7-64.4), while individuals who were seropositive at baseline (S1+) and were vaccinated by SVY1 maintained SARS-CoV-2 seroprevalence of >90% across all four surveys (Supplementary Figure 5). All participants that were S1+ and vaccinated had seroprevalence approaching 100% (97.3-100%) at SVY4 irrespective of timing of vaccination. For S1- participants, those who were vaccinated earlier maintained higher seropositivity over time.

There was substantial heterogeneity in the trajectory of antibody titers over time (Supplementary Figure 6). Among the 1000 participants that were COVID-19 vaccine-naïve throughout the study, the median normalized OD ratio remained low throughout the study in those that were S1- (-0.29 [SVY1] to 2.72 [SVY4]), while S1+ and vaccine-naïve individuals had comparatively higher titers, but remained lower than those of participants that were vaccinated at any timepoint (Supplementary Table 6; Supplementary Figure 7). Vaccinated participants that were S1+ had higher median OD ratios at all timepoints than S1- individuals, irrespective of the timing of first vaccination. Median OD ratios did not differ by the number of vaccine doses received. However, median OD ratios were higher among S1- individuals who had received two doses of AZ vaccines than those who had one dose of J&J vaccine in SVY3 and SVY4 (Supplementary Figure 8). There was no difference in antibody titers by vaccine type in S1+ individuals.

Sero-reversions were observed; among those that were S1+ and remained vaccine-naïve during the study (n = 369), 88 (23.9%, 95% CI 19.6-28.5%) became seronegative at SVY2 and 120 (32.5%, 95% CI 27.8-37.6%) at SVY3 (Supplementary Figure 5). As for S1+ individuals that were vaccinated by SVY1 (n = 65), 3.1% (0.3-10.7%; n = 2) were seronegative at SVY2, while 7.1% (2.0-17.3%; 4/56) of the same group were seronegative at SVY3.

## Discussion

Our prospective longitudinal serosurvey highlighted increasing SARS-CoV-2 seroprevalence in urban (Lilongwe) and rural (Karonga) communities through three significant waves of infection. Seropositivity was predominantly driven by natural infection, as confirmed by high seroprevalence among unvaccinated individuals. Seroprevalence was highest in participants 15-29 years at all timepoints, likely due to greater mobility and interactions with others. At SVY4, participants aged 30+ in Karonga who had received two doses of vaccine had significantly higher SARS-CoV-2 seroprevalence than unvaccinated persons. Otherwise, seroprevalence did not differ by COVID-19 vaccination status. Individuals with hybrid immunity (who had evidence of previous infection [seropositive] and had received vaccination) had higher seropositivity and higher antibody titers than (1) vaccine-naïve individuals and (2) individuals who were vaccinated but had no previous infection. Sero-reversions were observed more frequently in infected than vaccinated individuals.

By the end of the Omicron BA.1/2 wave, population SARS-CoV-2 seroprevalence was estimated at 89% in Karonga and 94% in Lilongwe. A cross-sectional community serosurvey estimated seroprevalence of 87.9% (95% CI 83.1-96.6%) in Lilongwe earlier in the Omicron wave [9]. A rural Uganda cohort reported a similar seroprevalence of 96% between February-March 2022 [17], while a cross-sectional serosurvey from Kenya between February-June 2022 reported slightly lower seroprevalence (Kilifi 69.1% [95% CrI 65.8-72.3]; Nairobi 88.5% [95% CrI 86.1-90.6%]) [18]. Unlike Malawi, both Uganda and Kenya imposed lockdowns at various times during the pandemic.

Applying our seroprevalence estimates to the national population of 20.4 million [19], the number of laboratory-confirmed COVID-19 cases (∼86,000 by end of April 2022) [4] suggest that less than 1% of SARS-CoV-2 infection were captured by national surveillance. SARS-CoV-2 serosurveys from other African settings have also reported considerable case under-ascertainment, with ratios of seroprevalence to cumulative confirmed case incidence ranging from 18:1 to 954:1 [20]. A substantial proportion (56-66%) of SARS-CoV-2 seropositive participants in our study were asymptomatic, with few requiring healthcare attendance or admission. The asymptomatic fraction in our study is likely an underestimate since the symptoms of COVID-19 substantially overlap with, and thus could be attributable to, other endemic infections such as malaria or other respiratory viruses.

Individuals with asymptomatic or mild SARS-CoV-2 infection have been shown to have lower antibody titers [21,22], earlier reductions in IgG and neutralizing antibody levels, as well as earlier sero-reversions, compared to symptomatic persons [21,22]. We observed a gradual decline in median OD ratios over successive surveys among those that were seropositive at SVY1 and never vaccinated and also identified sero-reversions, which occurred more frequently in individuals who were infected but unvaccinated, compared to vaccinated individuals. It is likely that a proportion of those vaccinated at SVY1 were infected before vaccination (i.e. have hybrid immunity), thus have stronger and more persistent antibody response.

Longitudinal sampling allowed us to demonstrate the contrasting dynamics of SARS-CoV-2 infection by geographical location and across age groups. The significantly higher seroprevalence and lower SARS-CoV-2 PCR positivity in Lilongwe compared to Karonga in SVY1 suggested that the Beta variant-driven wave occurred earlier and was more widespread in Lilongwe. This was consistent with greater population movement and transmission in the capital city during the festive period (before SVY1). Our modeling indicated that seroconversion risk was similar in the rural and urban sites over the subsequent waves. Sun et al. likewise described a higher infection attack rate within their urban cohort during the Beta-driven infection wave in South Africa, while the rural cohort had higher infection attack rates during the Delta and Omicron BA.1/2 waves [23].

Similar to other settings [20,24], SARS-CoV-2 seroprevalence in our cohort was consistently higher in adults (>15 years) than in children, even when restricted to unvaccinated individuals. Children were significantly less likely to be seropositive than adults at SVY1. However, their odds of seroconversion increased with successive surveys, with the largest increase between SVY3-4. By SVY4, those aged 0-14 years and 30+ years had similar seroprevalence. Aside from age and sex, increasing household size was the only other factor significantly associated with seroconversion, though only between SVY1-2. Seroconversion may be associated with household size since SARS-CoV-2 exposure within the household is likely to be in close contact, and more prolonged. Additionally, personal protective equipment such as face masks is unlikely to be used, compared to transient public exposures. A longitudinal serosurvey in rural Uganda reported no association between household characteristics and seroconversion [17]. However, the study noted within-household clustering of seroconversions, particularly between children. This suggests a high secondary attack rate in younger age groups within the household [17].

Few African studies have evaluated SARS-CoV-2 antibody responses longitudinally in the community [17,23,[25], [26], [27], [28], [29]]. A study in Ethiopia recruited cohorts of healthcare workers and community participants, but sampling occurred at different timepoints at different sites, and a high rate of loss-to-follow-up necessitated participant replacement at later surveys [27]. In Uganda, SARS-CoV-2 serology was performed on plasma collected from a malaria cohort, but less than half of the cohort contributed to all surveys [17]. The largest cohort study to date (PHIRST-C) has conducted 2-monthly serosurveys in 1200 individuals from a rural and an urban community in South Africa since July 2020, providing detailed characterization of SARS-CoV-2 infection and immunity across the four major waves [23,25,26,28]. Findings from PHIRST-C also highlighted the increasingly complex immunological landscape within the cohort, describing 14 categories of SARS-CoV-2 exposure histories after Omicron BA.1/2 wave and estimated that very few individuals (<6%) remain naïve to SARS-CoV-2 [23].

Aside from longitudinal sampling, key strengths of our study include prospective follow-up individuals from randomly selected households, ensuring that seroprevalence estimates reflected the immunological landscape of the broader population at the study sites. Inclusion of participants of all ages allowed us to characterize the varying seroconversion risk in different age categories. Additionally, we were able to demonstrate the absence of spatial clustering or hotspots of infection at either geographic location, suggesting that risk of SARS-CoV-2 infection outside the household was similar irrespective of participant occupation or sociodemographic characteristics.

Our study has several limitations. First, it focused on two geographic sites, which may not be representative of the general population of Malawi. Second, there was substantially greater loss-to-follow-up in the urban than the rural cohort. Higher attrition of urban participants has been previously observed [30]. Third, we did not capture all SARS-CoV-2 infections within the cohort during the study period, as follow-up took place 3-monthly, and only a proportion of participants had PCR tests. Fourth, our ELISA had a relatively low sensitivity of 86.5%. Of note, sensitivity estimates for commercial antibody assays are usually derived from convalescence samples of hospitalized patients with severe disease (in whom seroconversion is more likely). Applying sensitivity estimates derived from severe cases to the general population who predominantly have mild/asymptomatic infection (in whom seroconversion is less likely), may lead to an overestimation of assay sensitivity due to spectrum bias (where the performance of a diagnostic test may vary in different clinical settings) [31]. We used convalescent samples from Malawian individuals with predominantly asymptomatic infection as positive controls, which may explain the comparatively lower sensitivity than that reported for the same assay in a Scottish study [15]. Furthermore, we were unable to evaluate the impact of comorbidities, such as HIV and diabetes, on SARS-CoV-2 seroprevalence and antibody responses due to low numbers of individuals with those conditions. Lastly, we did not account for antibody waning and, therefore, will likely have underestimated cumulative seroprevalence.

## Conclusion

Our longitudinal sero-epidemiology study has provided a better understanding of the true extent of SARS-CoV-2 infection and varying transmission dynamics by age as well as urban/rural settings over the successive waves in Malawi. Akin to other African studies, we identified a high level of population SARS-CoV-2 exposure after the Omicron BA.1/2 wave despite low vaccination uptake (including significantly higher vaccine refusal in rural dwellers).

Our results have important implications for the national COVID-19 vaccination policy. High seroprevalence in the context of low COVID-19-related morbidity suggests that limited vaccine supplies could be targeted at vulnerable groups, rather than deploying them to the general population. This recommendation is made under the assumption that no significantly immune evasive and virulent new variant arises in the near future. Although we are unable to define high-risk groups from these data, ongoing work to characterize virus neutralization activity may highlight populations that are seropositive but do not generate a neutralizing antibody response and therefore are not protected against reinfection. A key unknown is how the current population immunity of Malawi, shaped by exposure to successive SARS-CoV-2 variants on the background of low vaccination rates, will impact the emergence of future variants. Lastly, ongoing disease and sero-surveillance studies encompassing both urban and rural populations are key to monitoring for emerging variants amidst high immune pressure, as a more pathogenic future variant may have significant impact in this low vaccine uptake setting.

## Declaration of Competing Interest

AH has received funding from UKRI, MRC, BSAC, Wellcome Trust, BSAC, and MRF unrelated to this work. JMR has received funding from UKRI (MR/V038613/1). The other co-authors have no conflicts of interest to declare.

## Appendix Supplementary materials

Image, application 1
