# Supplementary material for: Characterizing the evolving SARS-CoV-2 seroprevalence in urban and rural Malawi between February 2021 and April 2022: A population-based cohort study
Source: Int J Infect Dis. 2023 Dec;137:118–25. doi: 10.1016/j.ijid.2023.10.020 (PMC10695832; doi:10.1016/j.ijid.2023.10.020)
Supplement: Supplementary file 1 [file mmc1.docx]

**Supplementary material**

[Supplementary Methods 3](#_Toc146056502)

[*Study sites* 3](#_Toc146056503)

[*Household selection* 3](#_Toc146056504)

*[Ascertainment of medical and socio-economic history](#_Toc146056505)* [4](#_Toc146056505)

[*Sample collection* 6](#_Toc146056506)

[*Sample size calculation* 6](#_Toc146056507)

[*Modelling vaccination uptake and dosage* 7](#_Toc146056508)

[*Mixed effects modelling* 7](#_Toc146056509)

[*SARS-CoV-2 ELISA* 9](#_Toc146056510)

[Supplementary Tables 11](#_Toc146056511)

[Supplementary Table 1. COVID-19 vaccination status in COVSERO participants by location 11](#_Toc146056512)

[Supplementary Table 2. COVID-19 vaccination status in COVSERO participants by age group 12](#_Toc146056513)

[Supplementary Table 3. Model estimates for vaccination uptake at Survey 4 12](#_Toc146056514)

[Supplementary Table 4. SARS-CoV-2 PCR positivity by survey and study site 14](#_Toc146056515)

[Supplementary Table 5. Reported symptoms within past 14 days, by SARS-CoV-2 PCR status 15](#_Toc146056516)

[Supplementary Table 6. Crude, and population-weighted, test performance-adjusted SARS-CoV-2 anti-spike protein IgG seroprevalence by study site 18](#_Toc146056517)

[Supplementary Figures 21](#_Toc146056518)

[Supplementary Figure 1. Flow chart of screened and recruited households and participants 21](#_Toc146056519)

[Supplementary Figure 2. Map of Malawi, showing the location of the Karonga Health Demographic Surveillance Site and Area 25, Lilongwe 22](#_Toc146056520)

[Supplementary Figure 3. S1 IgG antibody responses for SARS-CoV-2 negative control and positive control samples 23](#_Toc146056521)

[Supplementary Figure 4. Semivariograms assessing spatial correlation between household-level initial seropositivity (Survey 1) and seroconversion between surveys, after accounting for age group, sex, occupational status and household size 24](#_Toc146056522)

[Supplementary Figure 5. Apparent SARS-CoV-2 seroprevalence in Surveys 1 to 4, stratified by vaccination status and baseline seropositivity 25](#_Toc146056523)

[Supplementary Figure 6. Trajectory of SARS-CoV-2 S1 IgG normalised OD ratios in participants that had attended all 4 study visits 27](#_Toc146056524)

[Supplementary Figure 7. Box and whiskers plot of median, IQR and range of SARS-CoV-2 S1 IgG normalised OD ratios stratified by baseline seropositivity 29](#_Toc146056525)

[Supplementary Figure 8. Box and whiskers plot of median, IQR and range of SARS-CoV-2 S1 IgG normalised OD ratios stratified by baseline seropositivity by vaccine type 31](#_Toc146056526)

[References 32](#_Toc146056527)

# **Supplementary Methods**

## ***Study sites***

MEIRU conducts population-based health research in rural and urban communities in Malawi. The rural study site, the Karonga Health and Demographic Surveillance System (Karonga HDSS) has provided health and demographic surveillance system information since 2002 [1] and a sampling frame for numerous population-based longitudinal health studies (citations needed). It is located in southern Karonga district, ~120km from the Tanzanian border, and the adult population is engaged predominately in subsistence farming and fishing.[1] In contrast, the urban site is a high-density urban residential area, called Area 25, in Lilongwe, where there is greater variation in socioeconomic status. The adult population is largely engaged in private and public sector employment [2]. Detailed existing individual and household-level data are available on residents in both areas and include demographic characteristics, socioeconomic status, family and household structures, geolocations and pre-existing co-morbidities (including HIV, hypertension and diabetes). These existing data provided an opportunity for in-depth analyses of risk factors and detailed sampling frames for selection of study participants.

## ***Household selection***

Households were selected using an inhibitory spatial design [3]. The households approached for inclusion in the study were selected at geographical coordinates determined by a modified randomised spatial inhibitory design with close pairs [4]. (Household locations were selected proportional to predicted population density according to existing geospatial data in both the urban and rural sites. The inhibitory design was used because it offered optimal spatial coverage with close pairs enabling detection of short- and long-range spatial correlation of disease prevalence.

The pairs of households enlisted for the study were identified using pre-determined GPS coordinates of the structures they inhabited, with “primary” households and “secondary” neighbourhood households. Households were randomly identified from existing lists from both the urban and rural sites. Generated lists included a designated “primary” household that was approached first.

Once the households were physically identified, informed consent, and assent for minors, was sought from inhabitants of each household member with the aim of recruiting a maximum of ten participants per household. Where more than ten people from a single household were willing to participate, household members were recruited in descending order of age. Upon recruitment of the primary household, the secondary household which would make up the pair could be approached from a list of three to four potential households in close proximity.

## ***Ascertainment of medical and socio-economic history***

**Co-morbidities.** History of diabetes mellitus, hypertension, asthma, chronic lung disease (not asthma), heart disease, chronic kidney disease, stroke, tuberculosis (past or present), chronic liver disease, human immunodeficiency virus (HIV), and cancer were ascertained by self-report of diagnosis and treatment by participant or by the parent/guardian of minors (<15 years).

**COVID-19 symptoms and healthcare attendance.** Participants were asked if they had experienced any severe headache, fatigue, fever, chills, runny or blocked nose, loss of smell, shortness of breath or difficulty breathing, wheeze, cough, nausea and/or vomiting, loss of appetite, loss of taste, diarrhoea, sore throat, chest pain or tightness, and muscle or joint pain since April 2020 at first study visit, and from the last visit in subsequent surveys. Any healthcare attendance and treatment relating to the above symptoms were also recorded.

**COVID-19 vaccine.** Receipt of COVID-19 vaccine, number of doses received, vaccine type, and date(s) of vaccination were recorded at every study visit.

**Education.** Highest level of education was ascertained from participant or parent or guardian of minors and categorised into i) never attended school; ii)) primary (1-5 years); iii) primary (6-8 years); iv) secondary; and v) tertiary.

**Occupation.** Employment type was categorised as i) unwaged; ii) irregular wage or piecework; or iii) regular waged or salaried.

**Household income.** Reported total household income in Malawian kwacha was categorised into quintiles by study location.

**Household asset index.** Participants were asked to report whether their household had the following possessions: working watch or clock, working radio, charcoal iron, working sewing machine, mobile phone, mosquito net, mattress, bed, bicycle, canoe, oxcart, motorcycle, car, in addition to electrical household items, including tape/CD player, fan, electric iron, television and refrigerator. A score based on the mean estimated value of the item in 2014 was generated for each individual item. A total asset index score was calculated for each participant, which we used to categorise households into quintiles of asset wealth by study location.

## ***Sample collection***

Nasal and throat swabs were self-collected by study participants using COVIDSafe Virus Inactivation Kit (ref: MS5760T3-50NOX1ML) with the aid of a mirror and MEIRU fieldworkers. Blood samples from were collected from adults (2ml) and minors (0.5ml) using BD vacutainer® 21G needles and 21G Vacucare auto safety lancets and put into Vacucare 2ml blood collection tubes with clot activator or Microvette®500 Z with clot activator, respectively. Samples were transported at 2-8°C and delivered to the local laboratory within four hours. Antibody and PCR test results were communicated to the participants via telephone or in person.

## ***Sample size calculation***

We calculated the power of the study to detect differences in antibody prevalence between sampling strata with 1,000 individuals in each stratum (e.g., different sites, or baseline compared to a follow-up). Any difference is conditional on the true prevalence in each sample (sample A and sample B) and test accuracy. We assumed test accuracy as above. If the true prevalence in sample A is 5%, we have 80% power to detect a sample B prevalence of <2.4% or >8.2% (at significance level of p<0.05). If sample A’s true prevalence is 10%, we are powered to detect prevalence <6.3% or >14.4% in sample B. We are powered to detect antibodies waning in 50 (100% test-positive) individuals if detectable antibody in follow-up sample <77%.

## ***Modelling vaccination uptake and dosage***

We fitted a multinomial model of the number of vaccination doses received (no vaccination, one dose, two doses) by Survey 4 with age group and study site as explanatory variables. The model was implemented using the *nnet* package in R.

## ***Mixed effects modelling***

Our model is described by the following equations:

$$y_{ij}\sim Bernoulli\left( p_{ij} \right)$$

$$p_{ij} = \pi_{ij}Se + \left( 1-\pi_{ij} \right)\left( 1-Sp \right)$$

$$\log it\left( \pi_{ij} \right)=\alpha+x_{ij}^{T}\beta+u_{j}$$

$$u_{j}\sim Normal\left( 0, \tau^{2} \right)$$

where $y_{ij}$ is the observed test result for individual $i$ in household $j$, $p$is the probability of observing that result given the test sensitivity $Se$and specificity, $Sp$, $x_{ij}$ are the fixed effects and $\beta$ their coefficients, and is the household-level random effect.$\mu_{j}$ is the household-level random effect. We used a Bayesian framework for parameter estimation, which was implemented as MCMC with the *nimble* package in R. 95% credible intervals (CrI) were computed to quantify uncertainty in posterior estimates.

For survey-specific estimates of seroprevalence, we modelled seropositivity with age group (0-14 years, 15-29 years, 30+ years) only as fixed effects for Survey (SVY) 1, and age group and vaccination dose received (0, 1, 2) as fixed effects for SVY4. We fitted models to each site independently. Very few individuals had received any vaccination at SVY1, hence we did not include vaccination dose as a fixed effect for SVY1. Few children (<15 years) had received vaccination by SVY4, so we excluded vaccinated children (<15 years) from the model for SVY4 due to insufficient observations. For SVY1, overall seroprevalence estimates for each site were calculated using predicted seropositivity by age group and census estimates of population size within each age group by site. For Survey 4, we combined model prediction of seropositivity by age group/vaccine dose for each site with probability estimates from an independent multinomial model of vaccination doses received, age group and site (see later in Supplement), to estimate the distribution of individuals in each age group/vaccination dose and thence the proportion within each category that were seropositive.

Using the same modelling framework, we estimated cumulative risk of seroconversion by modelling seroconversion among unvaccinated individuals at each survey who had tested negative in all previous surveys; for SVY1 we included all unvaccinated individuals. We estimated the cumulative risk for each age group within each site.

We identified risk factors for seroconversion due to infection between each survey using the mixed effects model, with additive categorical fixed effects of age group, sex, occupation type and study site, a linear fixed effect of household size, and a random intercept for household membership. We restricted observations to individuals that were unvaccinated and tested negative in all previous surveys; for SVY1, we used all individuals assuming that all were seronegative prior to the pandemic. Household-level random effects were tested for spatial correlation by constructing posterior empirical semivariograms, using distance bin-widths of 50 metres, and a maximum distance of three kilometres.

## ***SARS-CoV-2 ELISA***

SARS-CoV-2 recombinant S1 antigen ([www.nibsc.org](http://www.nibsc.org)) were immobilized to Immulon 2HB ELISA plates (ThermoFisher Scientific, UK) overnight at 4°C. Plates were washed three times with PBS/0.05% tween-20(v/v) [PBST] to remove unbound protein, blocked with the PBST plus 10% casein (PBSTC) at room temperature for one hour and washed. One positive serum sample and 5 negative sera (plasma samples from the MEIRU FIND study collected in January 2019; NHSRC protocol #16/09/1668) were included as controls in each run. Fifty-microliters (µL) samples or controls diluted 1:100 in PBSTC were tested in duplicate. After one hour of incubation at room temperature, plates were washed three times and 50µL of a horseradish peroxidase-labelled rabbit anti-human IgG (Bethyl laboratories via Cambridge Bioscience, UK), diluted 1:3000 was added. Plates were then incubated for 1 hour at room temperature, washed three times and 50 µL TMB (KPL, Gaithersburg, USA) was added to all wells and the mixture was incubated for 10 minutes in the dark. The reaction was stopped with 50 µL of 1M H_2_SO_4_ and optical density (OD) was read immediately at 450nm (OD) on a microplate reader (Accuris SmartReader 96; Benchmark Scientific, Edison, NJ, USA). Duplicate samples with >15% coefficient of variation were repeated.

As several studies had demonstrated lower specificity of SARS-CoV-2 ELISA assays in samples obtained from African studies [5, 6], we undertook further assay validation with 246 sera from a prospective cohort of Malawian adults between 2013 and 2015 as negative controls (COMREC protocol P.11/12/1310) [7], and 96 sera obtained from Malawian individuals >4 weeks from a positive SARS-CoV-2 PCR result as positive controls (COMREC protocol P.05/20/3053). S1 ELISA correctly classified 243 of 246 pre-pandemic sera samples as seronegative, while 83 of 96 sera from known SARS-CoV-2 PCR-positive individuals were seropositive, giving an assay sensitivity of 86.5% (95% confidence interval (CI) 79.6-93.3%) and specificity of 98.8% (95%CI 97.4-100%) (Supplementary Figure 2).

# **Supplementary Tables**

## **Supplementary Table 1. COVID-19 vaccination status in COVSERO participants by location**

| COVID-19 vaccine | Survey 1 | | Survey 2 | | | Survey 3 | | | Survey 4 | | |
| --- | --- | --- | --- | --- | --- | --- | --- | --- | --- | --- | --- |
|  | **Rural**  **Karonga**  **n=1005** | **Urban**  **Lilongwe**  **n=1000** | | **Rural**  **Karonga**  **n=952** | **Urban Lilongwe**  **n=721** | | **Rural**  **Karonga**  **n=941** | **Urban**  **Lilongwe**  **n=603** | | **Rural**  **Karonga**  **n=889** | **Urban**  **Lilongwe**  **n=531** |
| No | 965 (96·0) | 923 (92·3) | | 749 (78·7) | 603 (83·6) | | 673 (73·7) | 463 (76·9) | | 575 (64·7) | 385 (72·5) |
| Yes | 26 (2·6) | 76 (7·6) | | 102 (10·7) | 114 (15·8) | | 128 (14·0) | 136 (22·6) | | 159 (17·9) | 140 (26·4) |
| AstraZeneca – 1 dose | 25 (96·2) | 76 (100) | | 100 (75·3) | 86 (75·4) | | 40 (31·2) | 26 (19·1) | | 52 (32·7) | 27 (19·7) |
| AstraZeneca – 2 doses | 0 (0) | 0 (0) | | 19 (18·8) | 23 (20·2) | | 57 (44·4) | 77 (56·6) | | 64 (40·2) | 82 (59·9) |
| Johnson & Johnson | 0 (0) | 0 (0) | | 6 (5·9) | 5 (4·4) | | 30 (23·4) | 33 (24·3) | | 42 (26·4) | 28 (20·4) |
| Unknown | 1 (3·8) | 0 (0) | | 0 (0) | 0 (0) | | 1 (0·8) | 0 (0) | | 1 (0·6) | 0 (0) |
| Refused vaccine | 14 (1·4) | 1 (0·1) | | 101 (10·6) | 4 (0·6) | | 112 (12·3) | 4 (0·7) | | 155 (17·4) | 6 (1·1) |

## **Supplementary Table 2. COVID-19 vaccination status in COVSERO participants by age group**

| COVID-19 vaccine | Survey 1 | | Survey 2 | | | Survey 3 | | | Survey 4 | | |
| --- | --- | --- | --- | --- | --- | --- | --- | --- | --- | --- | --- |
|  | **15-29**  **n=583** | **>30**  **n=765** | | **15-29**  **n=455** | **>30**  **n=645** | | **15-29**  **n=402** | **>30**  **n=585** | | **15-29**  **n=364** | **>30**  **n=559** |
| No | 569 (97.6) | 662 (86.5) | | 383 (84.2) | 396 (61.3) | | 314 (78.1) | 291 (49.7) | | 250 (68.7) | 214 (38.3) |
| Yes | 10 (1.7) | 92 (12.0) | | 28 (6.1) | 188 (29.2) | | 42 (10.5) | 224 (38.4) | | 44 (12.1) | 255 (45.7) |
| AstraZeneca – 1 dose | 10 (1.7) | 92 (12.0) | | 26 (5.7) | 138 (21.4) | | 12 (3.0) | 56 (9.6) | | 18 (5.0) | 64 (11.5) |
| AstraZeneca – 2 doses | 0 (0) | 0 (0) | | 2 (0.4) | 41 (6.4) | | 15 (3.8) | 120 (20.5) | | 10 (2.7) | 136 (24.4) |
| Johnson & Johnson | 0 (0) | 0 (0) | | 0 (0) | 9 (1.4) | | 15 (3.8) | 48 (8.2) | | 16 (4.4) | 55 (9.8) |
| Refused vaccine | 4 (0.7) | 11 (1.4) | | 44 (9.7) | 61 (9.5) | | 46 (11.4) | 70 (12.0) | | 70 (19.2) | 90 (16.1) |

*No children <15 years received COVID-19 vaccination

## **Supplementary Table 3. Model estimates for vaccination uptake at Survey 4**

|  | Coefficient | Standard error | Adjusted odds ratio (95% CI) |
| --- | --- | --- | --- |
| 1 dose compared to unvaccinated |  |  |  |
| Intercept | -1·5041526 | 0·1657052 | - |
| Age (years) |  |  |  |
| 30+ (reference) | 0 | - | 1 |
| 0-14 | -13·071342 | 66·4860299 | 0·00 (0·000, Inf) |
| 15-29 | -1·320312 | 0·2790302 | 0·27 (0·16, 0·46) |
| Location |  |  |  |
| Karonga (reference) | 0 | - | 1 |
| Lilongwe | -0·1249393 | 0·2495644 | 0·88 (0·54, 1·44) |
| 2 doses compared to 1 dose |  |  |  |
| Intercept | -0·6934527 | 0·1224826 | - |
| Age (years) |  |  |  |
| 30+ (reference) | 0 | - | 1 |
| 0-14 | -5·027747 | 0·7146621 | 0·01 (0·00, 0·03) |
| 15-29 | -2·045430 | 0·2281442 | 0·13 (0·08, 0·20) |
| Location |  |  |  |
| Karonga (reference) | 0 | - | 1 |
| Lilongwe | 0·5044057 | 0·1687695 | 1·656 (1·19, 2·31) |

## **Supplementary Table 4. SARS-CoV-2 PCR positivity by survey and study site**

| Survey* |  | | SARS-CoV-2 PCR positivity | | |
| --- | --- | --- | --- | --- | --- |
|  |  | | **Karonga** | **Area 25 Lilongwe** | **p-value** |
| 1 |  | | 29/299 (9·7) | 2/279 (0·7) | 0·001 |
| 2 |  | | 48/497 (9·7) | 19/436 (4·4) | 0·002 |
| 3 |  | | 9/503 (1·8) | 4/365 (1·1) | 0·41 |
| 4 | | 9/506 (1·8) | | 4/332 (1·2) | 0·51 |

PCR, polymerase chain reaction

*50% adult participants were approached for self-collected nasal and throat swabs in Survey 1,

while all adult participants were approached in Surveys 2-4

## **Supplementary Table 5. Reported symptoms within past 14 days, by SARS-CoV-2 PCR status**

| Symptom | Survey 1 | | | Survey 2 | | | Survey 3 | | | Survey 4 | | |
| --- | --- | --- | --- | --- | --- | --- | --- | --- | --- | --- | --- | --- |
|  | **SARS-CoV-2 PCR Negative**  **N=547** | **SARS-CoV-2 PCR Positive**  **N=31** | **P-value** | **SARS-CoV-2 PCR Negative**  **N=866** | **SARS-CoV-2 PCR Positive**  **N=67** | **P-value** | **SARS-CoV-2 PCR Negative**  **N=854** | **SARS-CoV-2 Positive**  **N=13** | **P-value** | **SARS-CoV-2 PCR Negative**  **N=825** | **SARS-CoV-2 PCR Positive**  **N=13** | **P-value** |
| Any COVID-19 symptoms | 216 (39·5) | 15 (48·4) | 0·33 | **379 (43·8)** | **46 (68·7)** | <0·001 | 307 (36·0) | 3 (23·1) | 0·34 | 345 (41·8) | 6 (46·2) | 0·75 |
| Fever | **32/546 (5·9)** | **5 (16·1)** | 0·04 | **52 (6·0)** | **11 (16·4)** | 0·004 | 49/852 (5·7) | 0 (0) |  | 74 (9·0) | 3 (23·1) | 0·11 |
| Fatigue | **32 (5·9)** | **6 (19·4)** | 0·01 | **43/865 (5·0)** | **10/67 (14·9)** | 0·003 | 49 (5·8) | 0 (0) |  | 50/823 (6·1) | 2 (15·4) | 0·19 |
| Chills/shivers | 40 (7·3) | 5 (16·1) | 0·08 | **62 (7·2)** | **15 (22·4)** | <0·001 | 36 (4·2) | 0 (0) |  | 70 (8·5) | 3 (23·1) | 0·10 |
| Headache | 74 (13·5) | 5 (16·1) | 0·60 | 132 (15·2) | 14 (20·9) | 0·223 | 124 (14·5) | 0 (0) |  | 148 (17·9) | 3 (23·1) | 0·71 |
| Myalgia | 16/546 (2·9) | 3 (9·7) | 0·07 | **43 (5·0)** | **10 (14·9)** | 0·003 | 48/853 (5·6) | 0 (0) |  | **44 (5·3)** | **4 (30·8)** | 0·005 |
| Arthralgia | 32 (5·9) | 4 (12·9) | 0·12 | 45/865 (5·2) | 6 (9·0) | 0·26 | 48 (5·6) | 0 (0) |  | **52 (6·3)** | **3 (23·1)** | 0·05 |
| Cough | 32 (5·9) | 4 (12·9) | 0·12 | **91/865 (10·5)** | **15 (22·4)** | 0·008 | 55/852 (6·5) | 0 (0) |  | 96/824 (11·6) | 2 (15·4) | 0·66 |
| Shortness of breath | **10 (1·8)** | **4 (12·9)** | 0·005 | 31/865 (3·6) | 6 (9·0) | 0·11 | 19 (2·2) | 0 (0) |  | 37/823 (4·5) | 2 (15·4) | 0·15 |
| Chest pain | 16/545 (2·9) | 3 (9·7) | 0·08 | 30/864 (3·5) | 3 (4·5) | 0·76 | 28 (3·3) | 0 (0) |  | 33 (4·0) | 2 (15·4) | 0·10 |
| Sore throat | 8 (1·5) | 4 (12·9) | 0·002 | 17/865 (2·0) | 4 (6·0) | 0·13 | 18/853 (2·1) | 0 (0) |  | 18 (2·2) | 1 (7·7) | 0·26 |
| Wheeze | 9 (1·7) | 1 (3·2) | 0·43 | 13/863 (1·5) | 4 (0·06) | 0·06 | 7 /852 (0·8) | 0 (0) |  | 18/824 (2·2) | 1 (7·7) | 0·26 |
| Runny nose | 56 (10·2) | 7 (22·6) | 0·07 | **142 (16·4)** | **29 (43·3)** | <0·001 | 67 (7·9) | 3 (23·2) | 0·08 | 106 (12·9) | 3 (23·1) | 0·23 |
| Loss of smell | 13/546 (2·4) | 2/30 (6·5) | 0·18 | **46/864 (5·4)** | **12 (17·9)** | 0·001 | 25 (2·9) | 0 (0) |  | 37 (4·5) | 1 (7·7) | 0·46 |
| Loss of taste | 5/546 (0·9) | 1 (3·2) | 0·28 | **35 (4·0)** | **8 (11·9)** | 0·009 | 32 (3·8) | 0 (0) |  | 39/823 (4·7) | 2 (15·4) | 0·15 |
| Nausea ± vomiting | 18 (3·3) | 2 (6·5) | 0·29 | 28/864 (3·2) | 5 (7·5) | 0·08 | 40 (4·7) | 0 (0) |  | 40 (4·9) | 1 (7·7) | 0·48 |
| Loss of appetite | 8/546 (1·5) | 2 (6·5) | 0·10 | **26/864 (3·0)** | **8 (11·9)** | 0·002 | 27 (3·2) | 0 (0) |  | 38 (4·6) | 2 (15·4) | 0·12 |
| Diarrhoea | **13 (2·4)** | **3 (9·7)** | 0·05 | 31 (3·6) | 3/67 (4·5) | 0·73 | 57 (6·7) | 0 (0) |  | 46/824 (5·6) | 1 (7·7) | 0·53 |

PCR, polymerase chain reaction

Denominator is as per top of column unless stated otherwise

## **Supplementary Table 6. Crude, and population-weighted, test performance-adjusted SARS-CoV-2 anti-spike protein IgG seroprevalence by study site**

|  | Karonga (rural) | | | | Lilongwe (urban) | | | |
| --- | --- | --- | --- | --- | --- | --- | --- | --- |
| Age group (years) | **Total**  **samples** | **Seropositive samples** | **Crude seroprevalence**  **% (95% CI)** | **Bayesian population-weighted, test-adjusted seroprevalence***  **% (95% CrI)** | **Total samples** | **Seropositive samples** | **Crude seroprevalence**  **% (95% CI)** | **Bayesian population-weighted, test-adjusted seroprevalence***  **% (95% CrI)** |
| Survey 1 |  |  |  |  |  |  |  |  |
| All | 1001 | 336 | 33·6 (30·6-36·6) | 26·3 (20·7-32·1) | 948 | 488 | 51·5 (48·2-54·7) | 46·4 (40·2-53·0) |
| <15 | 372 | 75 | 20·2 (16·2-24·6) | 14·1 (8·8-20·4) | 276 | 83 | 30·1 (24·7-35·9) | 24·3 (17·4-33·2) |
| 15-29 | 281 | 116 | 41·3 (35·4-47·3) | 35·8 (25·4-46·0) | 277 | 166 | 59·9 (53·9-65·8) | 59·3 (48·5-70·2) |
| >30 | 348 | 145 | 41·7 (36·4-47·0) | 36·0 (31·6-40·1) | 395 | 239 | 60·5 (55·5-65·3) | 59·9 (55·5-64·1) |
| Survey 2 |  |  |  |  |  |  |  |  |
| All | 947 | 326 | 34·4 (31·4-37·6) | - | 715 | 386 | 54·0 (50·2-57·7) | - |
| <15 | 361 | 73 | 20·2 (16·4-24·7) | - | 216 | 79 | 36·6 (30·1-43·4) | - |
| 15-29 | 258 | 102 | 39·5 (33·5-45·8) | - | 191 | 112 | 58·6 (51·3-65·7) | - |
| >30 | 328 | 151 | 46·0 (40·6-51·6) | - | 308 | 195 | 63·3 (57·7-68·7) | - |
| Survey 3 |  |  |  |  |  |  |  |  |
| All | 910 | 404 | 44·4 (41·1-47·7) | - | 600 | 338 | 56·3 (52·3-60·4) | - |
| <15 | 350 | 120 | 34·3 (29·3-39·5) | - | 187 | 80 | 42·8 (35·6-50·2) | - |
| 15-29 | 244 | 115 | 47·1 (40·7-53·6) | - | 152 | 88 | 57·9 (49·6-65·9) | - |
| >30 | 316 | 169 | 53·4 (47·8-59·1) | - | 261 | 170 | 65·1 (59·0-70·9) | - |
| Survey 4¨ |  |  |  |  |  |  |  |  |
| All | 886 | 650 | 73·4 (70·3-76·3) | 89·3(85·4-92·2) | 531 | 429 | 80·8 (77·2-84·1) | 93·9 (90·6-96·5) |
| <15 | 338 | 212 | 62·7 (57·3-67·8) | 85·2 (81·6-88·3) | 165 | 112 | 67·9 (60·2-74·9) | 91·4 (88·1-94·6) |
| 15-29 | 234 | 195 | 83·3 (77·9-87·9) | 95·4 (90·7-98·2) | 125 | 109 | 87·2 (80·0-92·5) | 96·7 (91·3-99·0) |
| >30 | 314 | 243 | 77·4 (72·4-81·9) | 89·9 (83·0-94·3) | 241 | 208 | 86·3 (81·3-90·4) | 94·6 (89·3-97·8) |

CI, confidence interval; CrI, credible interval.

*Prevalence estimates were calculated by using multilevel regression and poststratification (MLRP) to account for differences in the sample population and the national population and subsequently adjusted for assay sensitivity and specificity.

¨Bayesian population-weighted estimates account for vaccination.

# **Supplementary Figures**

## **Supplementary Figure 1. Flow chart of screened and recruited households and participants**

**
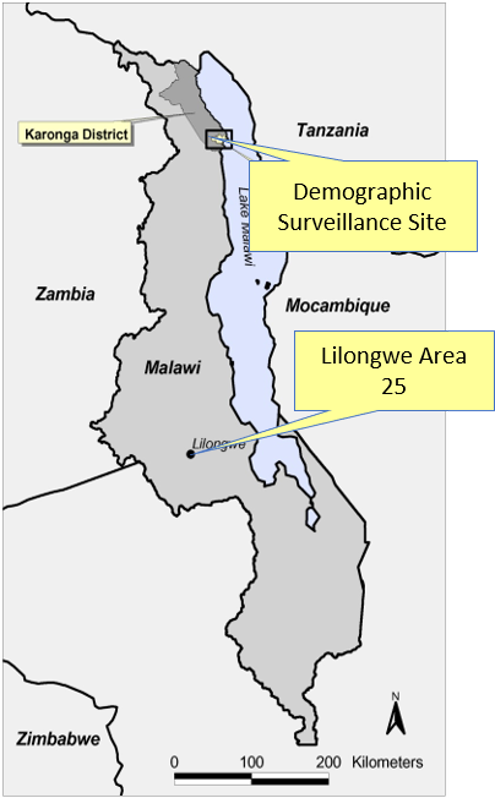
**

## **Supplementary Figure 2. Map of Malawi, showing the location of the Karonga Health Demographic Surveillance Site and Area 25, Lilongwe**


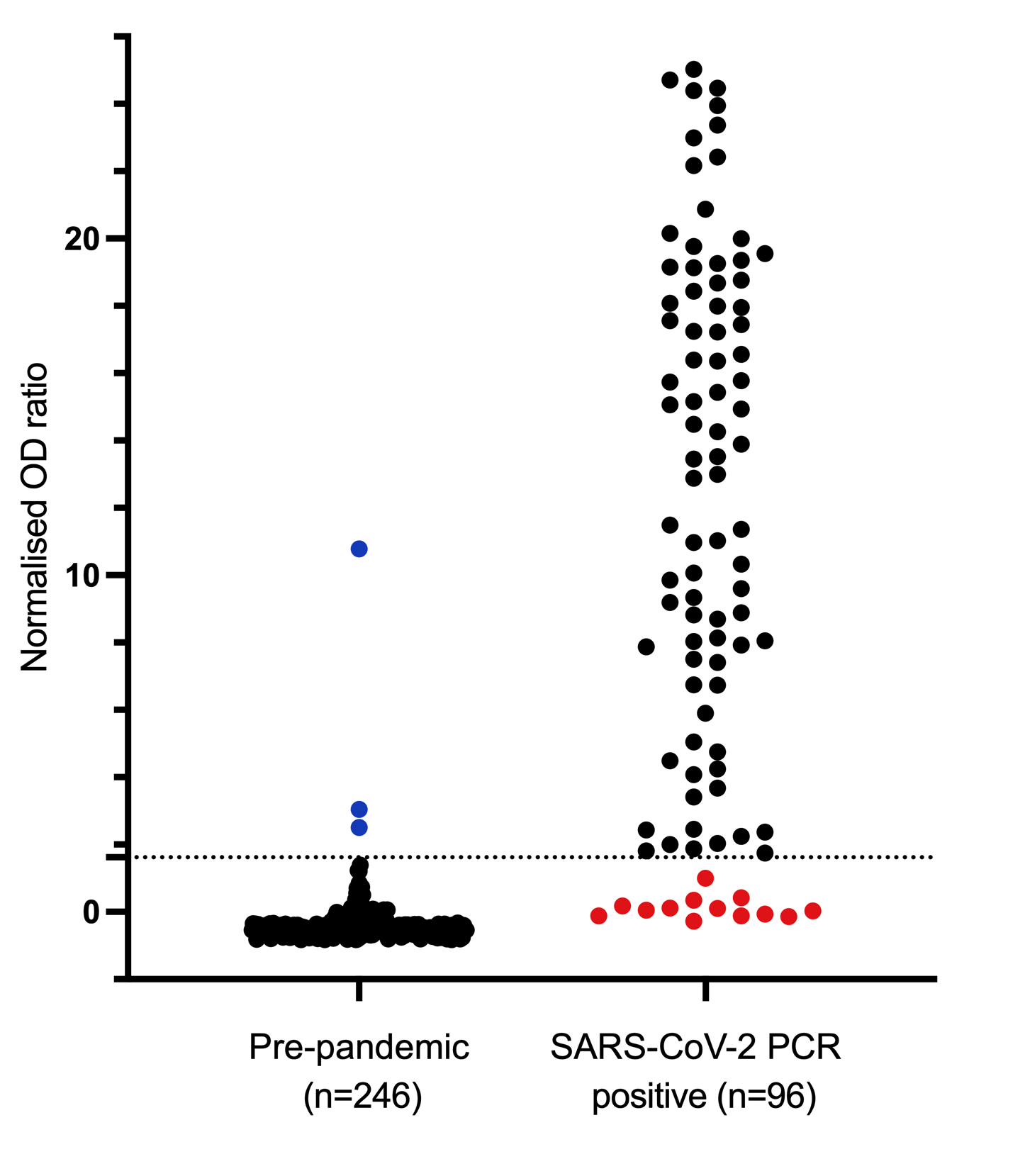


**Supplementary Figure 3. S1 IgG antibody responses for SARS-CoV-2 negative control and positive control samples.** The cut-off for seropositivity (normalised optical density (OD) ratio 1·620) shown in the dashed line. Abbreivations: PCR, polymerase chain reaction.

**
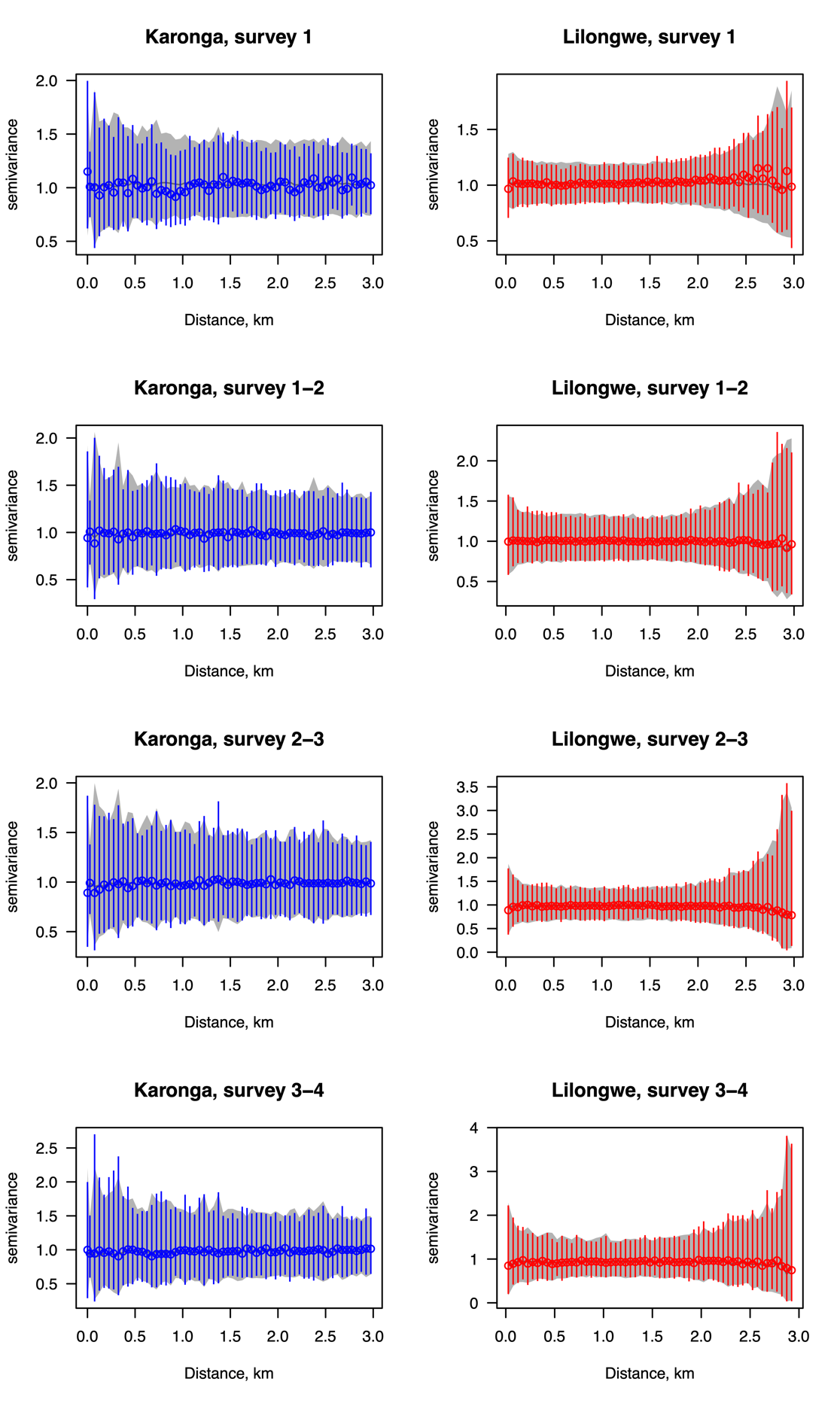
**

**Supplementary Figure 4. Semivariograms assessing spatial correlation between household-level initial seropositivity (Survey 1) and seroconversion between surveys, after accounting for age group, sex, occupational status and household size**. Coloured points and lines represent the mean and 95% credible intervals of variance as each distance; dark grey lines and grey regions represent the variance from a null model with households reassigned locations (from sampled households) at random.

**Supplementary Figure 5. Apparent SARS-CoV-2 seroprevalence in Surveys 1 to 4, stratified by vaccination status and baseline seropositivity.**

V0, never received COVID-19 vaccination; V1, received 1 or more COVID-19 vaccines at Survey 1**;** V2, received 1 or more COVID-19 vaccines at Survey 2; V3, received 1 or more COVID-19 vaccines at Survey 3; V4, received 1 or more COVID-19 vaccines at Survey 4; S1-, SARS-CoV-2 S1 IgG negative at baseline; S1+, SARS-CoV-2 S1 IgG positive at baseline.

| 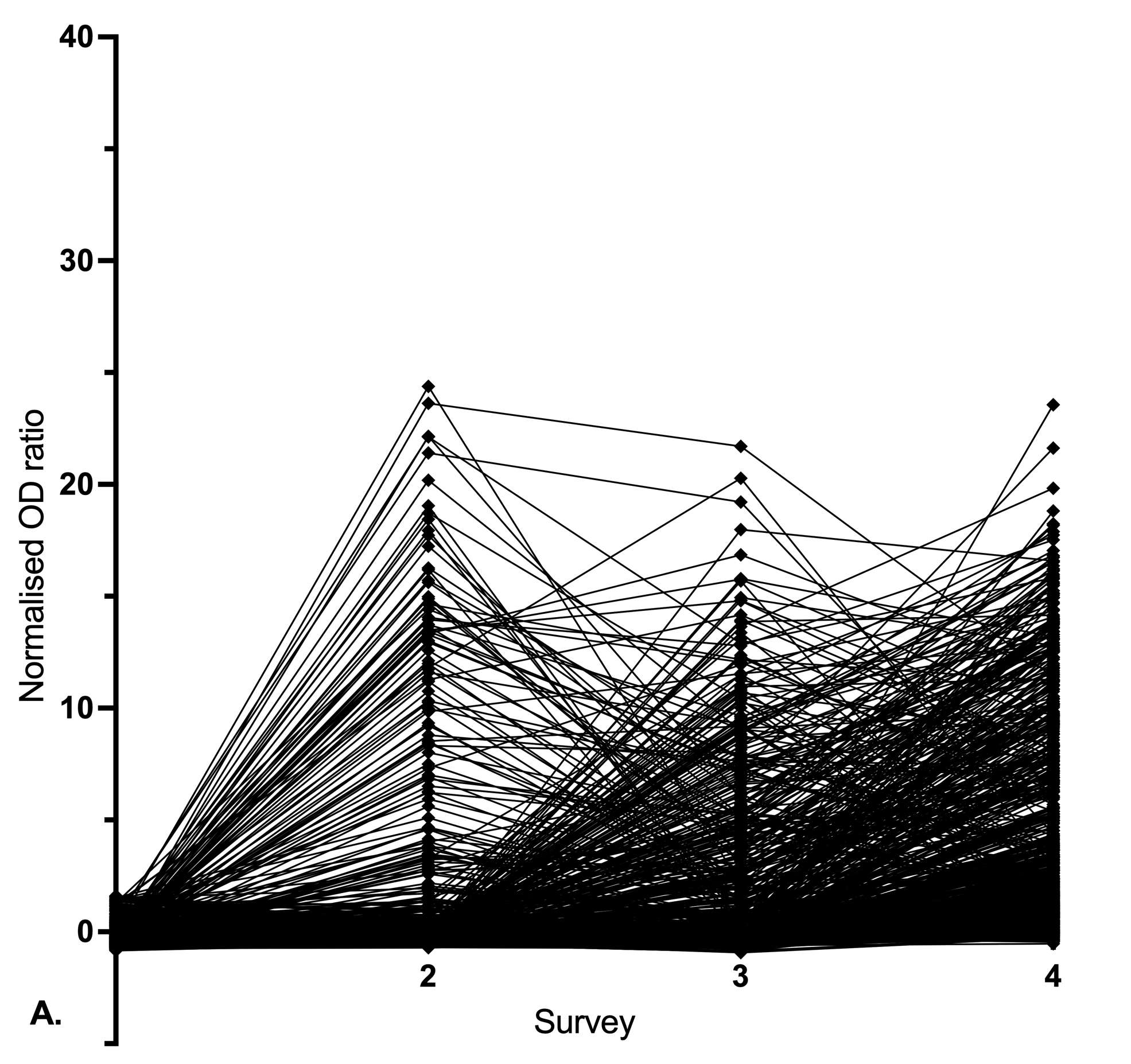 | 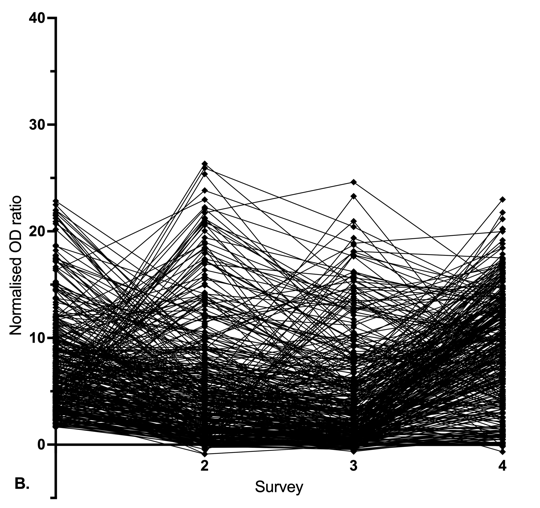 | 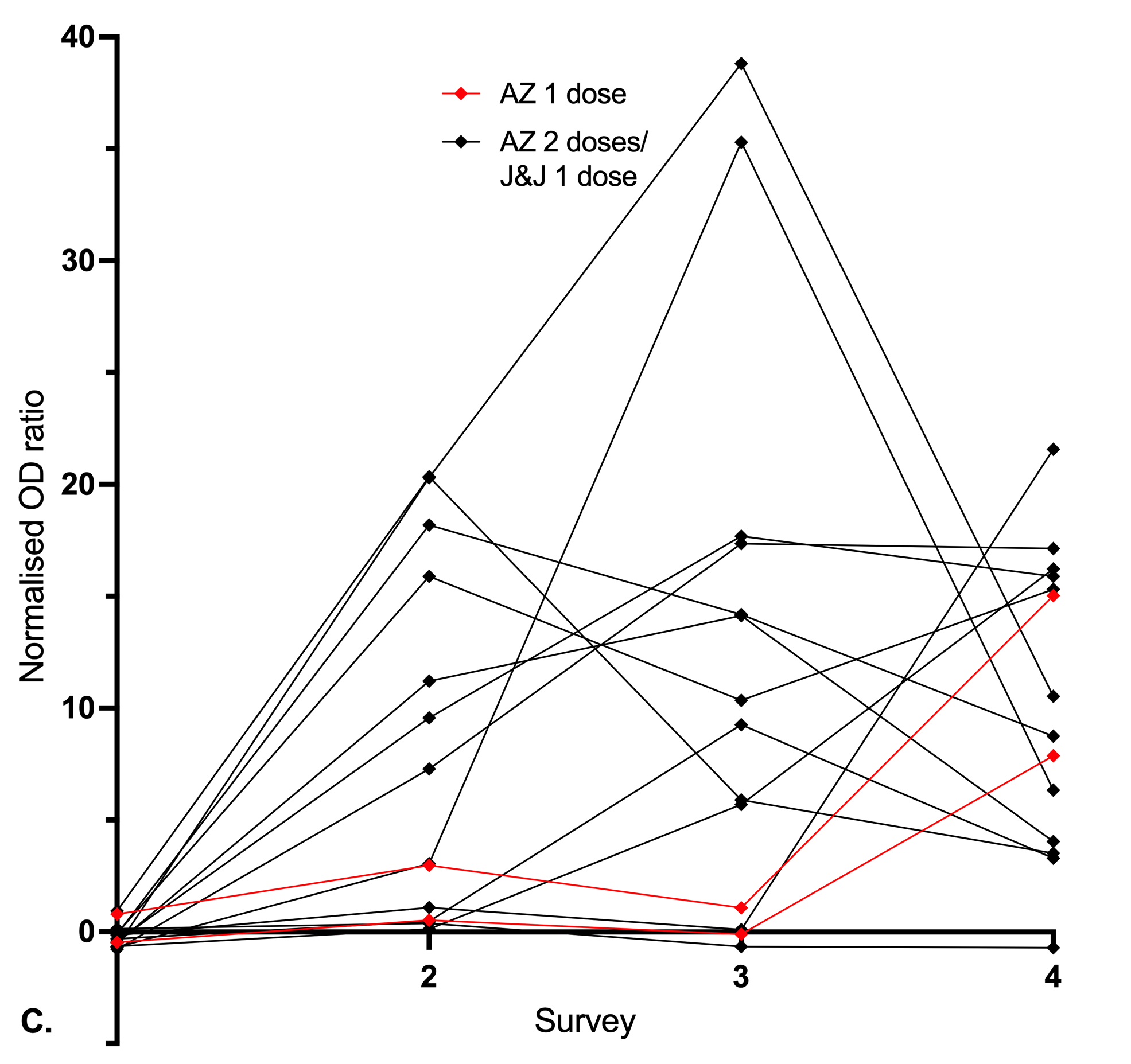 | 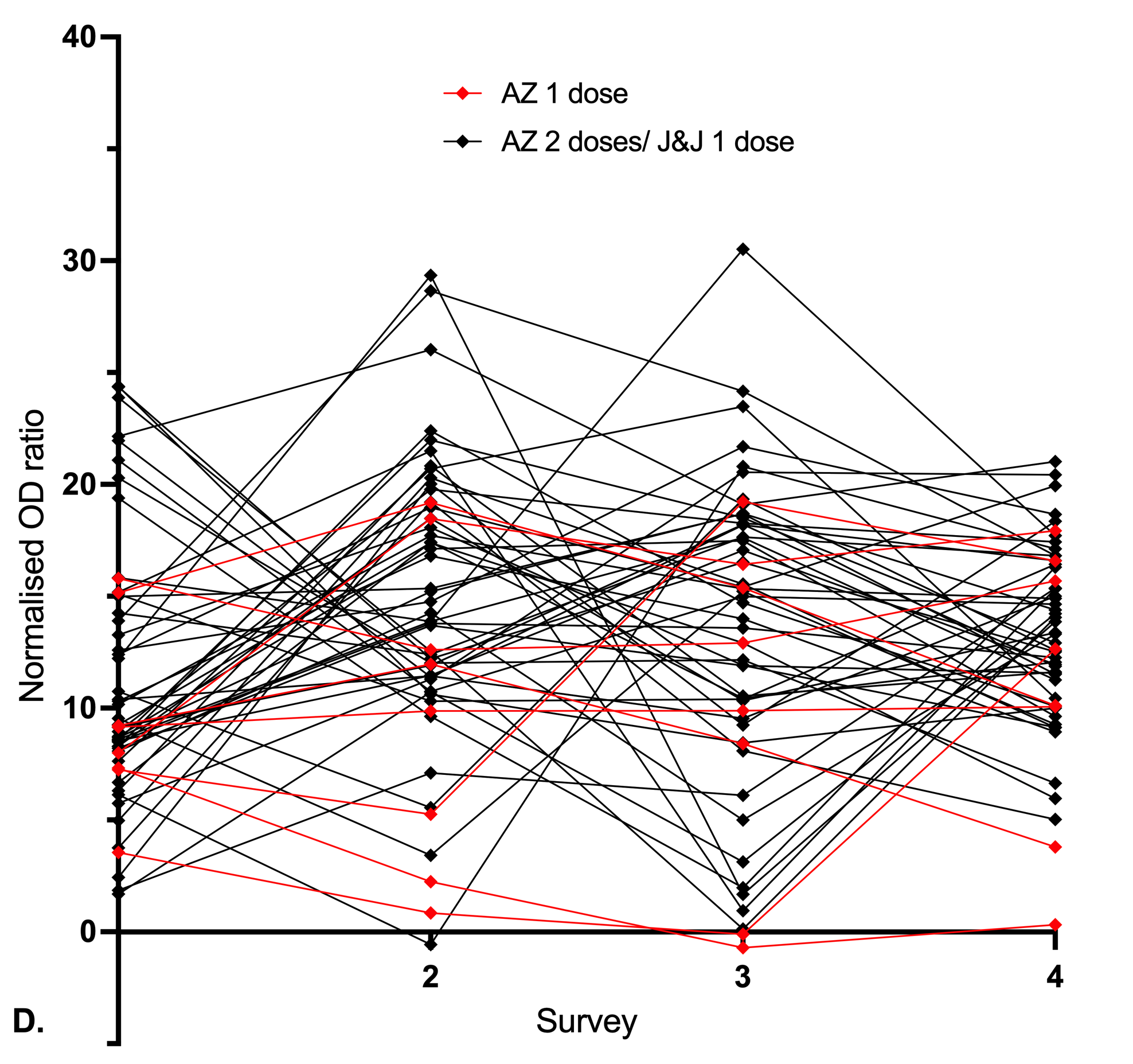 |
| --- | --- | --- | --- |
| 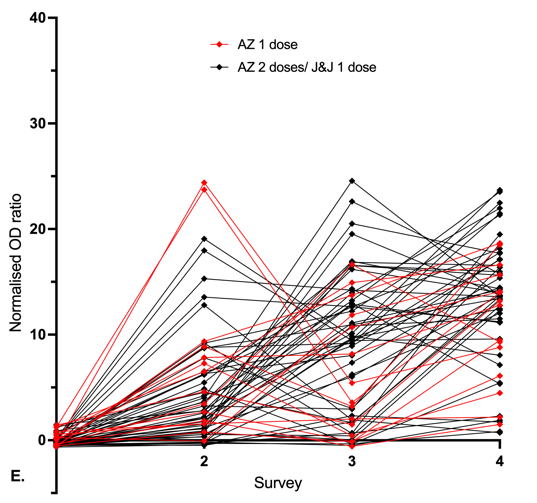 | 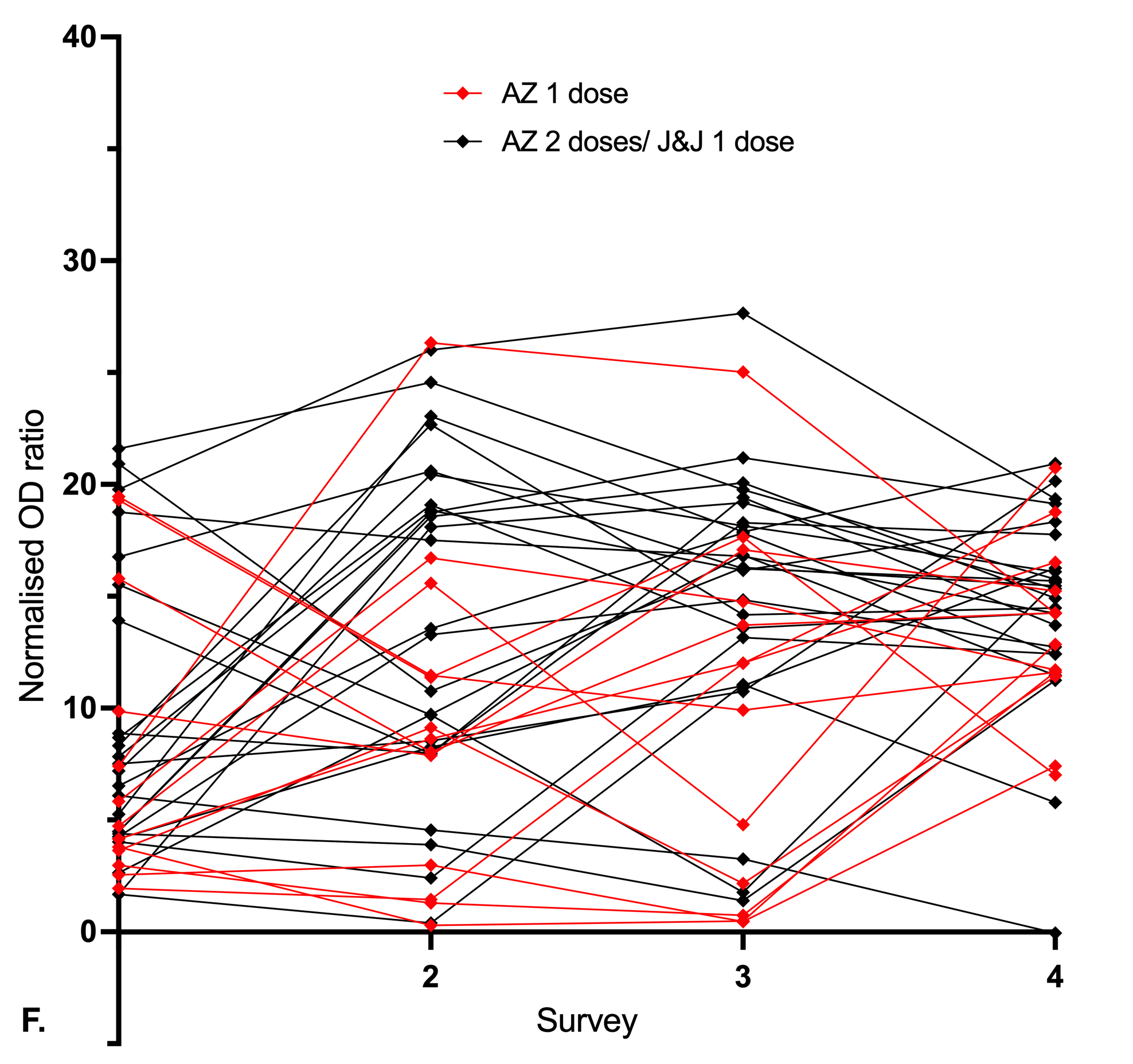 | 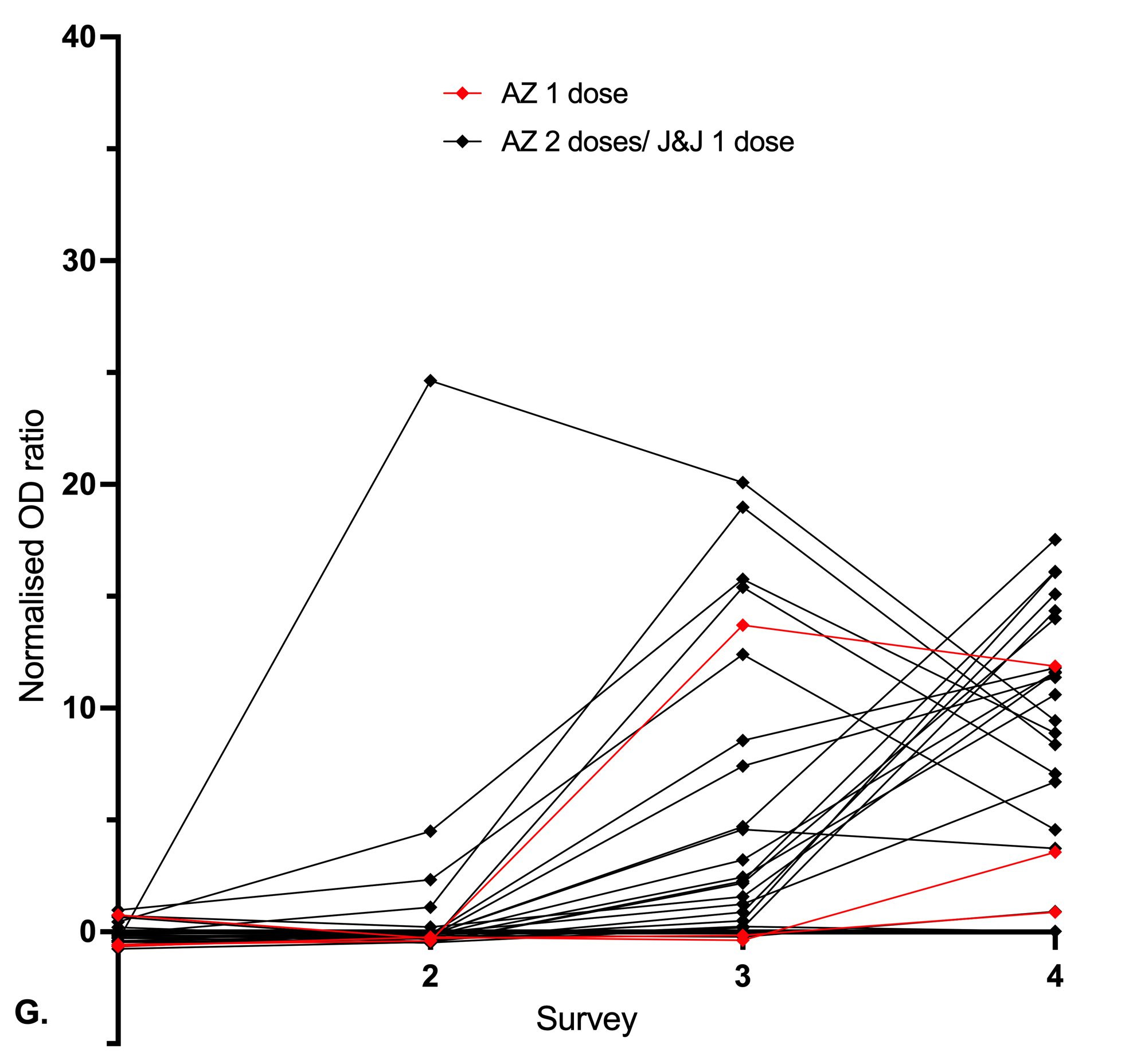 | 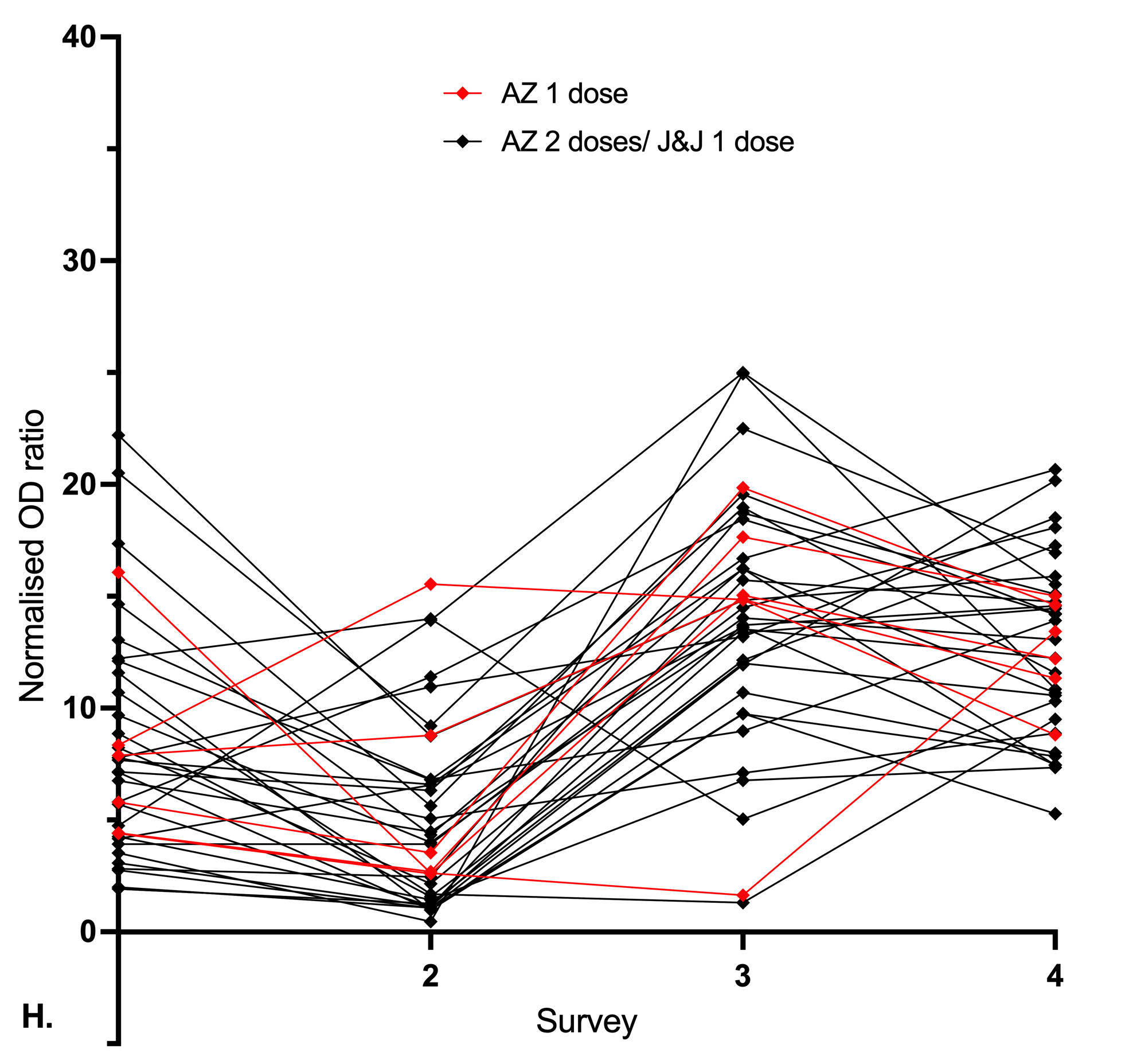 |
| 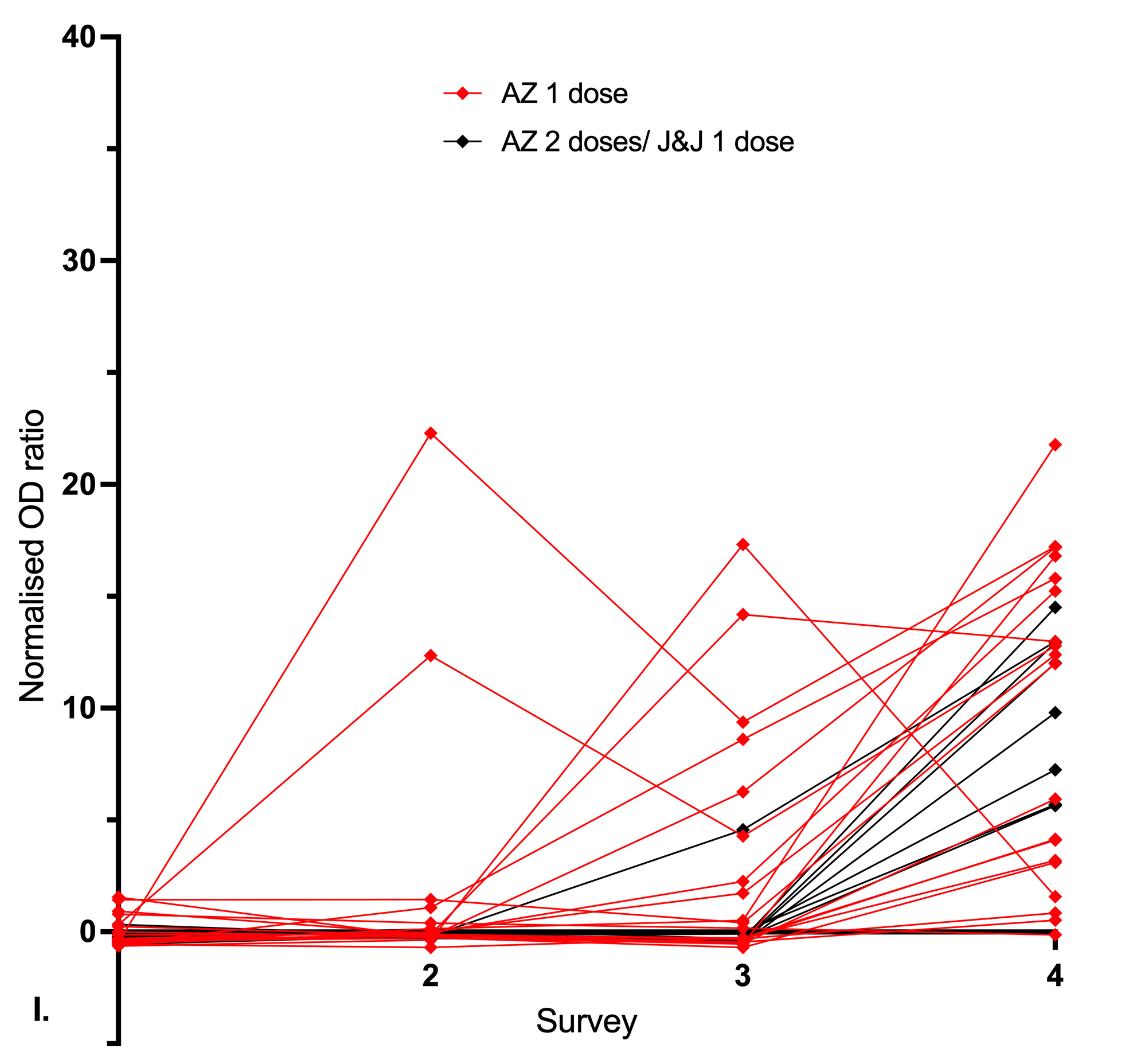 | 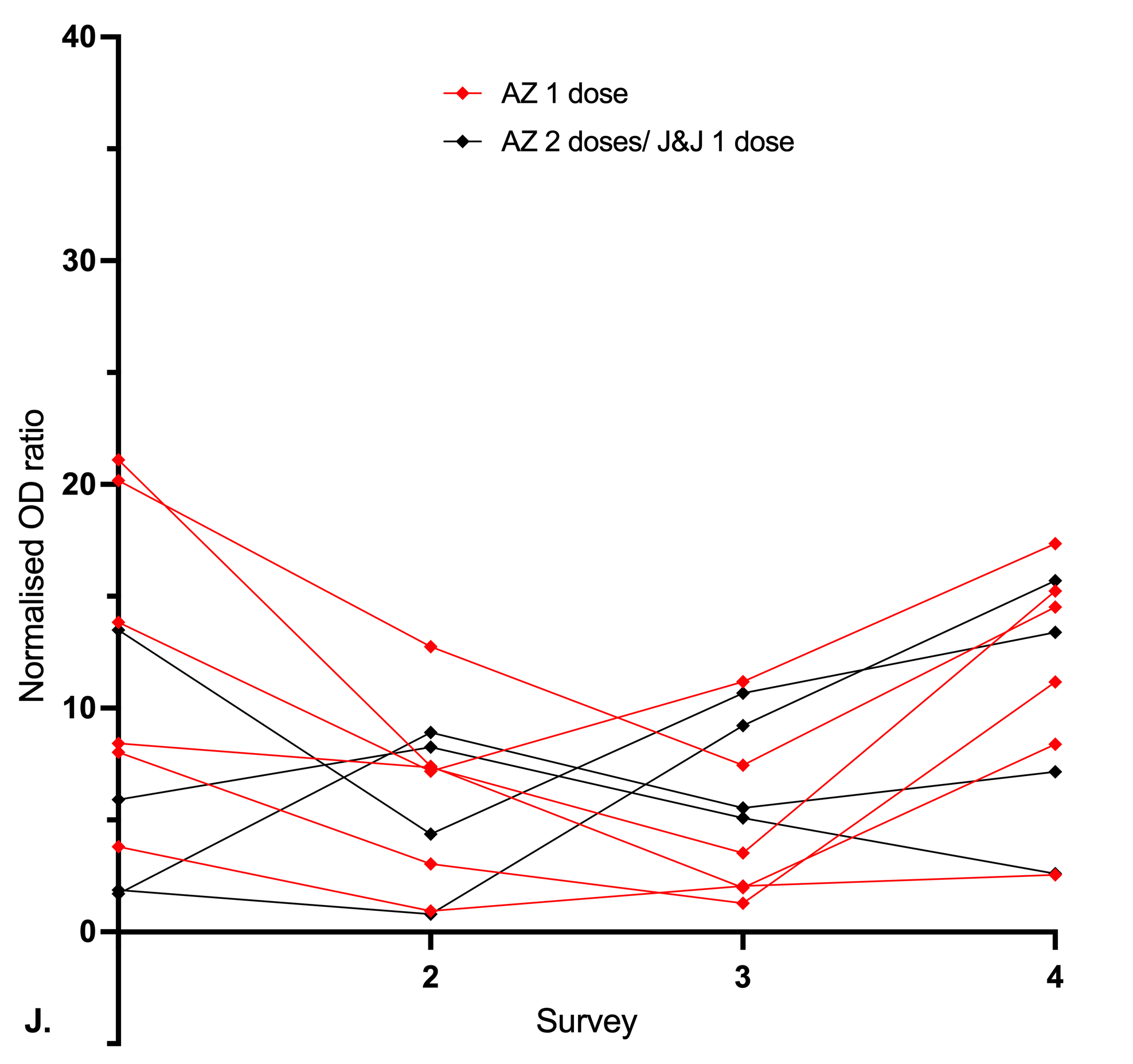 | **Supplementary Figure 6. Trajectory of SARS-CoV-2 S1 IgG normalised OD ratios in participants that had attended all 4 study visits** that were **A)** SARS-CoV-2 S1 IgG negative at baseline and has never received COVID-19 vaccine (n=650); **B)** SARS-CoV-2 S1 IgG positive at baseline and has never received COVID-19 vaccine (n=350); **C)** SARS-CoV-2 S1 IgG negative at baseline and had received >1 COVID-19 vaccine at baseline (n= 14); **D)** SARS-CoV-2 S1 IgG positive at baseline and had received first COVID-19 vaccine at baseline (n= 54); **E)** SARS-CoV-2 S1 IgG negative at baseline and had received first COVID-19 vaccine by survey 2 (n= 62); **F)** SARS-CoV-2 S1 IgG negative at baseline and had received first COVID-19 vaccine by survey 2 (n= 38); **G)** SARS-CoV-2 S1 IgG negative at baseline and had received >1 COVID-19 vaccine by survey 3 (n= 23); **H)** SARS-CoV-2 S1 IgG positive at baseline and had received first COVID-19 vaccine by survey 2 (n= 36); **I)** SARS-CoV-2 S1 IgG negative at baseline and had received >1 COVID-19 vaccine by survey 4 (n= 27); **J)** SARS-CoV-2 S1 IgG positive at baseline and had received first COVID-19 vaccine by survey 4 (n=10) | |

| **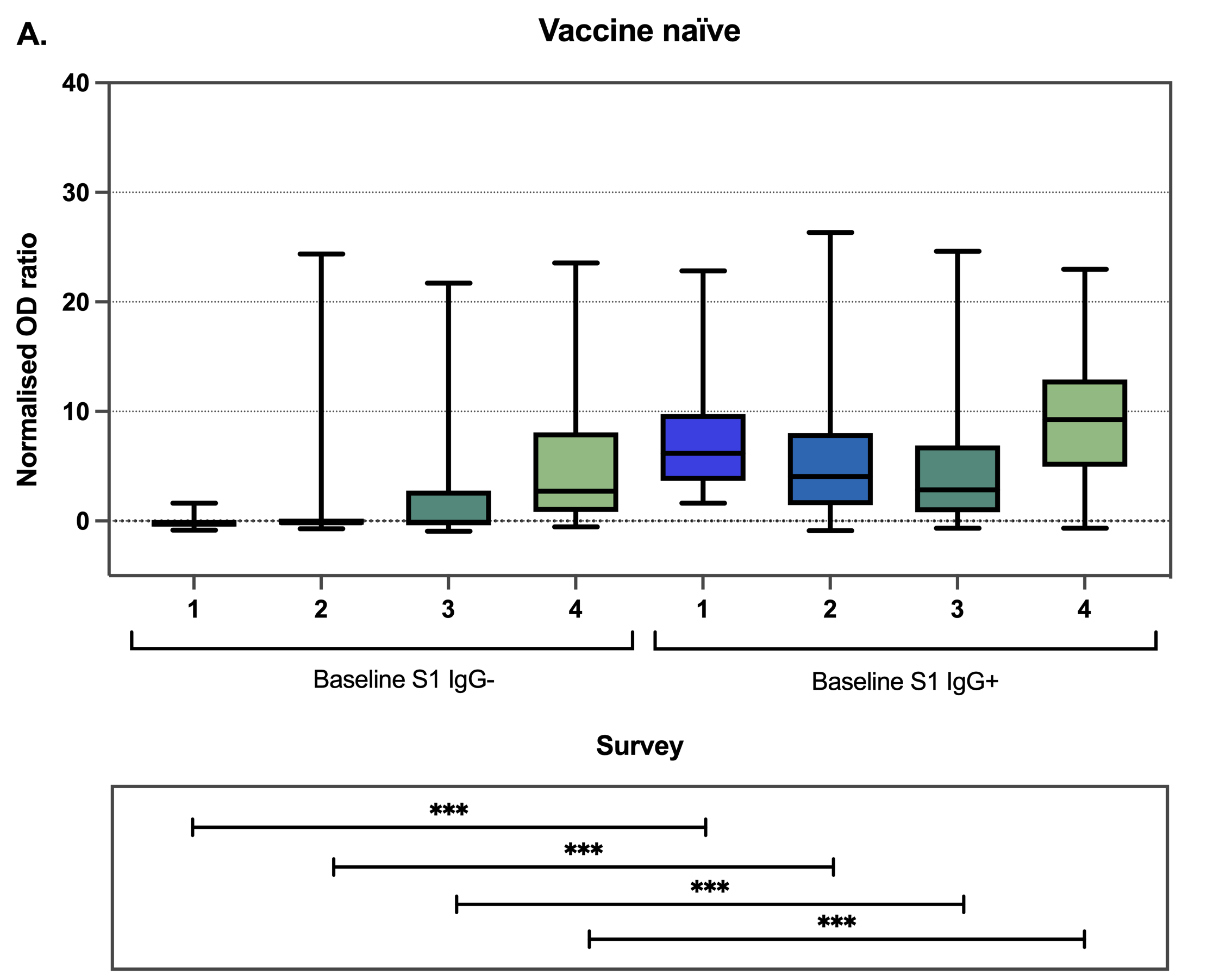** | **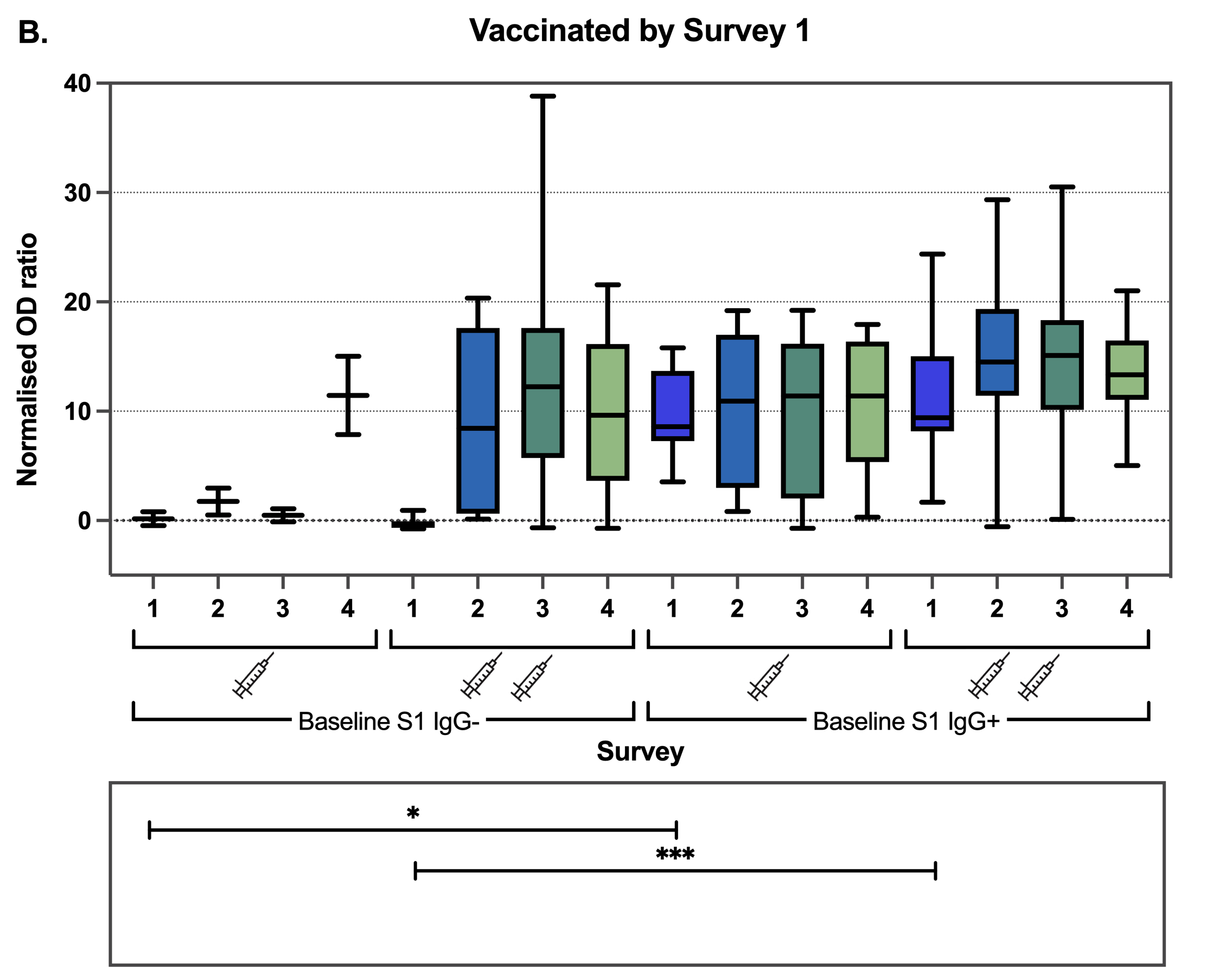** |
| --- | --- |
| **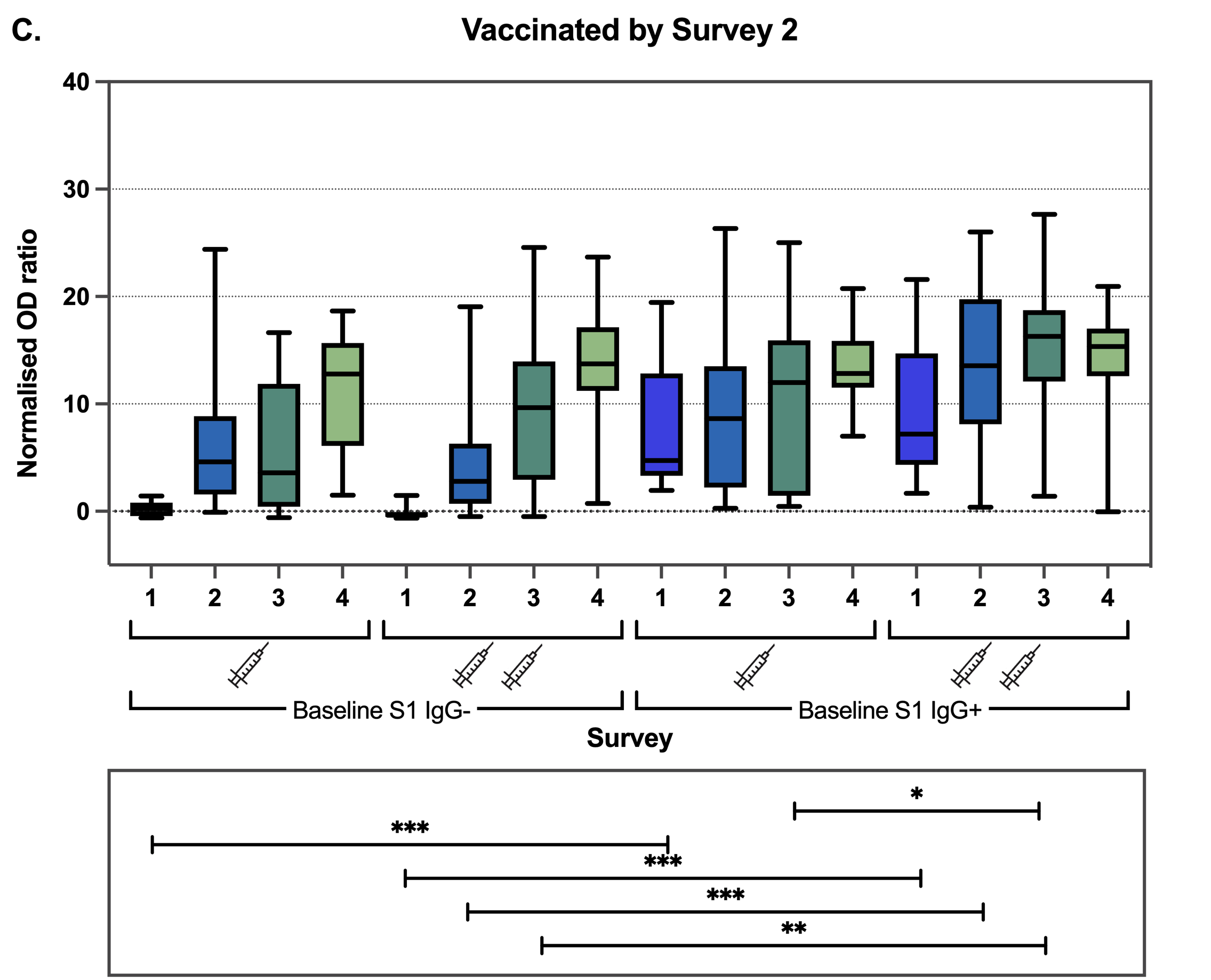** | **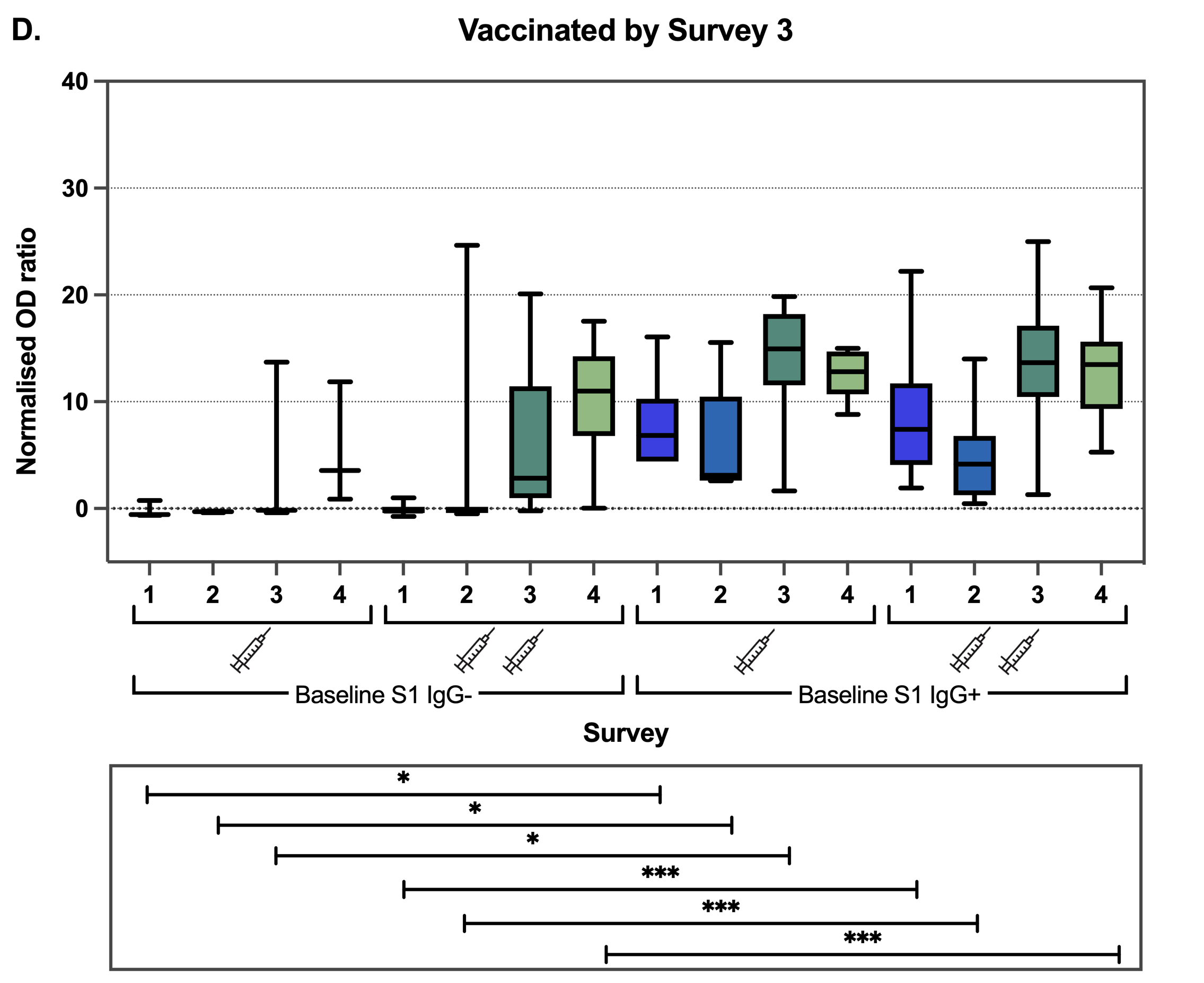** |
| **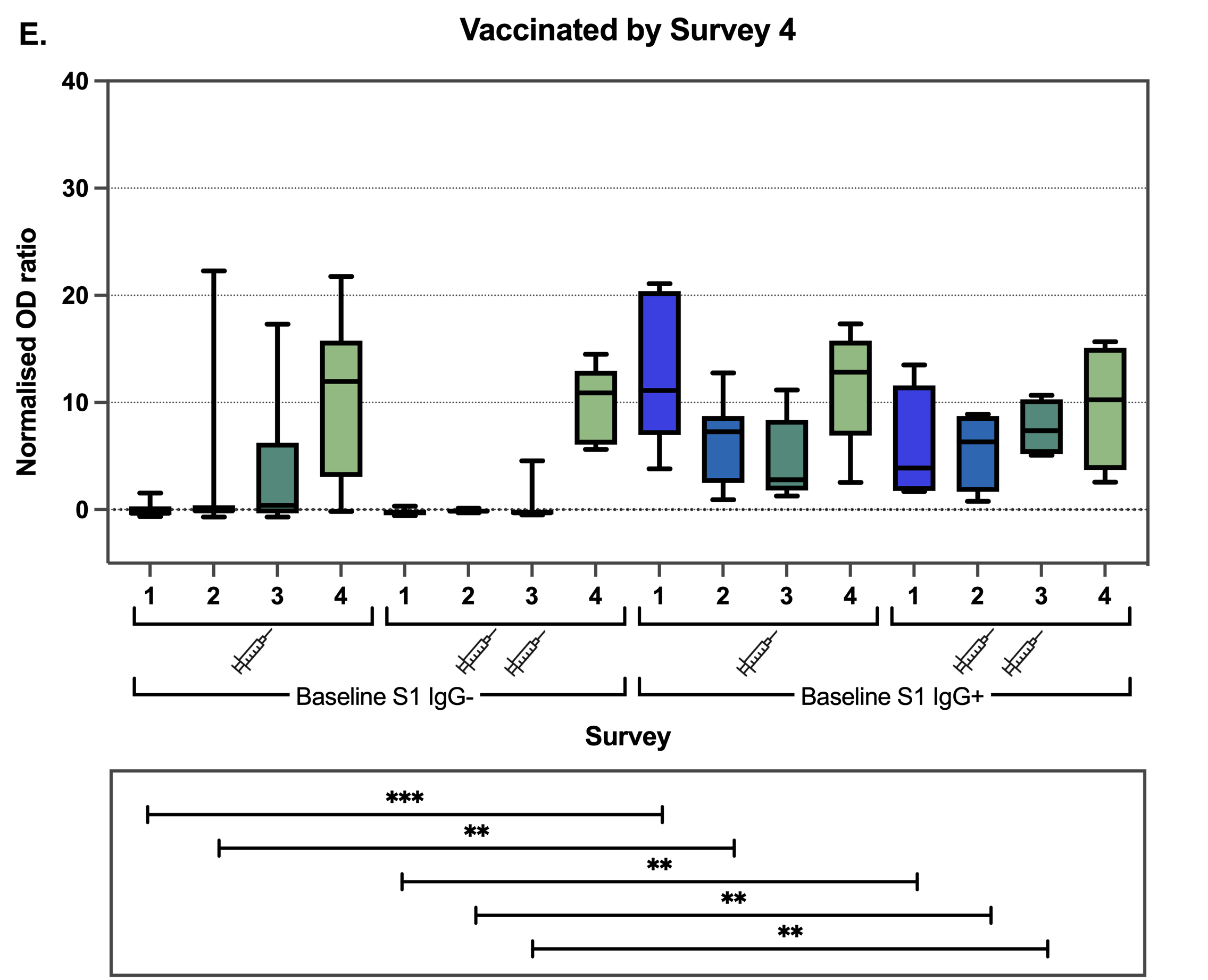** | **Supplementary Figure 7. Box and whiskers plot of median, IQR and range of SARS-CoV-2 S1 IgG normalised OD ratios stratified by baseline seropositivity in A)** participants that have never received COVID-19 vaccination; **B)** participants had reported receiving 1 or more COVID-19 vaccines at Survey 1**; C)** participants had reported receiving 1 or more COVID-19 vaccines at Survey 2; **D)** participants had reported receiving 1 or more COVID-19 vaccines at Survey 3; **E)** participants had reported receiving 1 or more COVID-19 vaccines at Survey 4. Box and whiskers plot: Survey 1, purple; Survey 2, blue; Survey 3, dark green; Survey 4, light green. *<0.05 **<0.001 ***<0.0001 |


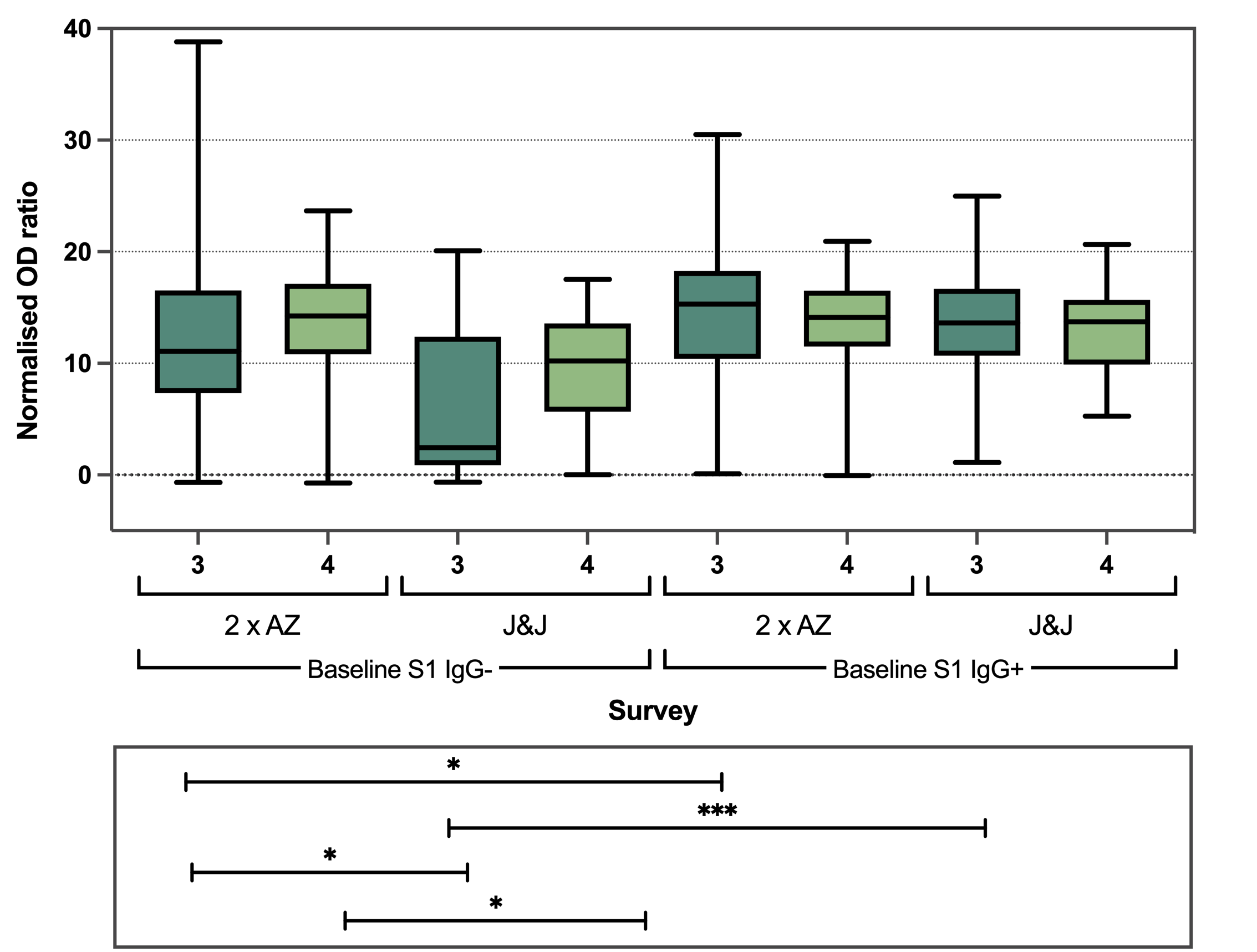


**Supplementary Figure 8. Box and whiskers plot of median, IQR and range of SARS-CoV-2 S1 IgG normalised OD ratios stratified by baseline seropositivity by vaccine type.** N.B. there were insufficient number of individuals that had received 2 doses of AZ vaccines or 1 dose of J&J vaccine in Surveys 1 and 2 for comparison.

AZ, AstraZeneca vaccine; J&J, Johnson & Johnson vaccine. Box and whiskers plot: Survey 3, dark green; Survey 4, light green. *<0.05 **<0.001 ***<0.0001

# **References**

[1] A. C. Crampin *et al.*, "Profile: The Karonga Health and Demographic Surveillance System," *International Journal of Epidemiology,* vol. 41, no. 3, pp. 676-685, 2012, doi: 10.1093/ije/dys088.

[2] A. J. Price *et al.*, "Prevalence of obesity, hypertension, and diabetes, and cascade of care in sub-Saharan Africa: a cross-sectional, population-based study in rural and urban Malawi," (in eng), *Lancet Diabetes Endocrinol,* vol. 6, no. 3, pp. 208-222, Mar 2018, doi: 10.1016/s2213-8587(17)30432-1.

[3] M. Chipeta, A. D. Terlouw, K. S. Phiri, and P. Diggle, "Inhibitory geostatistical designs for spatial prediction taking account of uncertain covariance structure.," *Environmetrics,* vol. 28, no. 1, p. e2425, 2017, doi: <https://doi.org/10.1002/env.2425>.

[4] M. G. Chipeta, D. J. Terlouw, K. S. Phiri, and P. J. Diggle, "Adaptive geostatistical design and analysis for prevalence surveys," *Spatial Statistics,* vol. 15, pp. 70-84, 2016/02/01/ 2016, doi: <https://doi.org/10.1016/j.spasta.2015.12.004>.

[5] A. Nkuba Ndaye *et al.*, "Challenges in interpreting SARS-CoV-2 serological results in African countries," *Lancet Glob Health,* vol. 9, no. 5, pp. e588-e589, May 2021, doi: 10.1016/S2214-109X(21)00060-7.

[6] P. Emmerich *et al.*, "Limited specificity of commercially available SARS-CoV-2 IgG ELISAs in serum samples of African origin," *Trop Med Int Health,* vol. 26, no. 6, pp. 621-631, Jun 2021, doi: 10.1111/tmi.13569.

[7] A. Ho *et al.*, "Impact of Human Immunodeficiency Virus on the Burden and Severity of Influenza Illness in Malawian Adults: A Prospective Cohort and Parallel Case-Control Study," *Clin Infect Dis,* vol. 66, no. 6, pp. 865-876, Mar 5 2018, doi: 10.1093/cid/cix903.
